# Sources and geographic origin of particulate matter in urban areas of the Danube macro-region: The cases of Zagreb (Croatia), Budapest (Hungary) and Sofia (Bulgaria)

**DOI:** 10.1016/j.scitotenv.2017.11.092

**Published:** 2018-04-01

**Authors:** M.G. Perrone, S. Vratolis, E. Georgieva, S. Török, K. Šega, B. Veleva, J. Osán, I. Bešlić, Z. Kertész, D. Pernigotti, K. Eleftheriadis, C.A. Belis

**Affiliations:** a Department of Earth and Environmental Sciences, University of Milano-Bicocca, P.zza della Scienza 1, 20126 Milan, Italy; b N.C.S.R. Demokritos, 15341 Ag. Paraskevi, Attiki, Greece; c National Institute of Meteorology and Hydrology, Bulgarian Academy of Sciences, 66 Blvd Tzarigradsko chaussee, 1784 Sofia, Bulgaria; d Centre for Energy Research, Hungarian Academy of Sciences, Konkoly Thege Miklos Utca 29-33, 1121 Budapest, Hungary; e Institute for Medical Research and Occupational Health, Ksaverska cesta 2, p.p. 291, 10001 Zagreb, Croatia; f Institute for Nuclear Research, Hungarian Academy of Sciences, Bem square 18/c, 4026 Debrecen, Hungary; g European Commission, Joint Research Centre, via Fermi 2749, I-21027 Ispra, VA, Italy

**Keywords:** Source apportionment, Long-range transport, PMF, FLEXPART, PSCF, Danube macro-region

## Abstract

The contribution of main PM pollution sources and their geographic origin in three urban sites of the Danube macro-region (Zagreb, Budapest and Sofia) were determined by combining receptor and Lagrangian models. The source contribution estimates were obtained with the Positive Matrix Factorization (PMF) receptor model and the results were further examined using local wind data and backward trajectories obtained with FLEXPART. Potential Source Contribution Function (PSCF) analysis was applied to identify the geographical source areas for the PM sources subject to long-range transport. Gas-to-particle transformation processes and primary emissions from biomass burning are the most important contributors to PM in the studied sites followed by re-suspension of soil (crustal material) and traffic. These four sources can be considered typical of the Danube macro-region because they were identified in all the studied locations. Long-range transport was observed of: a) sulphate-enriched aged aerosols, deriving from SO_2_ emissions in combustion processes in the Balkans and Eastern Europe and b) dust from the Saharan and Karakum deserts. The study highlights that PM pollution in the studied urban areas of the Danube macro-region is the result of both local sources and long-range transport from both EU and no-EU areas.

## Highlights

• The Danube Region encompasses one of Europe's air pollution “hot spots”.
• Causes of PM pollution were studied in the pilot cities: Zagreb, Budapest and Sofia.
• PM sources and their geographical origin were estimated using PMF and FLEXPART.
• Main PM sources in the Danube are aged aerosols, biomass burning, soil and traffic.
• Long-range transport of PM from non-EU areas is a key issue in the Danube Region.

## Graphical abstract

**Figure:**
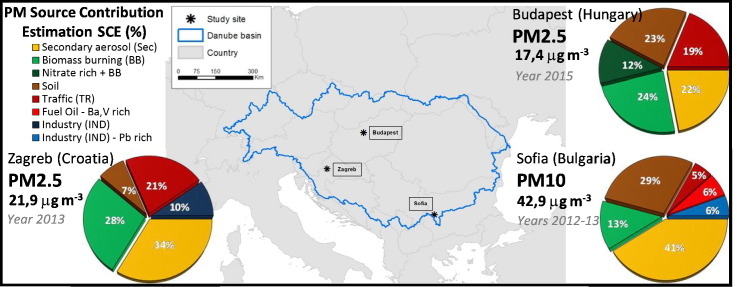

## 1 Introduction

Particulate matter (PM) is among the most harmful atmospheric pollutants in highly populated areas, and projections foresee outdoor PM is likely to cause even more severe health effects in the future unless more stringent policies are adopted (EEA, 2015a, OECD, 2016). Such impact of PM pollution has been associated with the high share (ca. 75%) of the European urban population exposed to annual PM_10_ concentrations that exceed the World Health Organization (WHO) air quality guideline concentrations (EEA, 2015a). The economic costs due to premature deaths caused by air pollution in Europe (UNECE region) have been estimated to be EUR 1000 billion, while those due to illness caused by air pollution, add up to EUR 100 billion (WHO, 2015).

The Danube macro-region encompasses one of Europe's air pollution “hot spots” (EEA, 2015b). The exceedances of PM_10_ or precursor gases, such as NO_2_ and SO_2_, have led to infringement procedures in almost all of the EU-Danube Member States and some of them have been referred to court. For that reason, controlling PM has become a challenge for the competent authorities. In order to design effective reduction strategies, quantitative information on the impact of sources on an area (source apportionment) is required.

Despite the increasing number of studies targeted at identifying and quantifying the typical sources of PM in many areas of Europe (Viana et al., 2008, Fragkou et al., 2012, Karagulian and Belis, 2012) there is still limited information about the sources of this pollutant in many areas of South-Eastern Europe (Belis et al., 2013). Moreover, pollution levels in the Danube macro-region are expected to be influenced to a varying extent by long-range transport of pollutants emitted beyond the EU (EEA, 2016). The approach adopted in this study has been to take advantage of existing datasets and to expand them, when necessary, with additional samples or chemical analyses. On the basis of the identification of the most critical areas and the availability of datasets, urban background sites representing different situations within the Danube macro-region were chosen as case studies. The aim of this work is to quantify the average contribution of emission sources to the ambient PM concentrations in the abovementioned sites and to identify their geographic origin with a view to support the development of effective emission abatement measures in the urban areas of the Danube macro-region.

## 2 Material and methods

### 2.1 Study area and PM sampling strategies

**Fig. 1:**
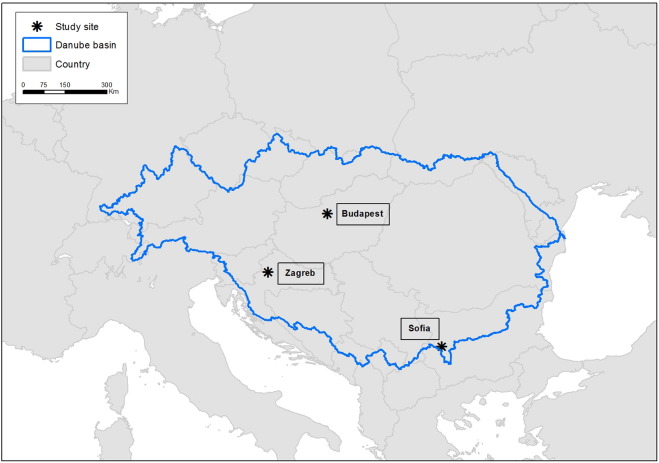
Location of the sampling sites in the Danube macro-region: Zagreb (ZGR), Budapest (BDP) and Sofia (SOF).

In this study filter-based PM samples collected in urban background (UB) sites in three cities of the Danube macro-region were used (Fig. 1): Zagreb (ZGR, Croatia), Budapest (BDP, Hungary) and Sofia (SOF, Bulgaria). BDP is located in a continental flat area, ZGR in a hilly area close to the Adriatic Sea, and SOF at the southern rim of the Danube basin. A summary of the monitoring sites and data collection strategy is given in Table 1. More details about the monitoring sites are provided in the Supplementary Material. Every dataset included PM mass and chemical composition, gaseous pollutants (NO_2_, SO_2_, CO and O_3_) and meteorological data (wind speed and direction, temperature and precipitation). PM daily concentrations were obtained in ZGR and SOF by gravimetric analysis (EN 12341). In BDP daily PM concentrations were obtained using an equivalent method (beta-ray absorption).

**Table 1:** The site locations, the time period (W = winter, SP = spring, SU = summer; F = fall) and the PM sampling in the three cities of the Danube macro-region.

City, Nation (Code)	*Sampling site* (latitude; longitude). Site type; short description	Time period (season: W SP SU F, year)	PM sampling (N)^a^	PM mass measuring
Zagreb, Croatia (ZRG)	*Ksaverska cesta* (45°50′07.87″ N; 15°58′38.86″ E). Urban background (UB); residential area in the Northside of ZGR	01 Jan 2013 to 31 Dec 2013 (all year, 2013)	PM10, PM2.5^b^, PM1; daily, 24 h, noon to noon, LVS (38,3 L/min). PM2.5 collected on Quartz filters (365).	Gravimetric
Budapest, Hungary (BDP)	*Gilice ter* (47°25′40.35″ N; 19°11′11.03″ E). Urban background (UB); residential area in the Southside of BDP	09Feb 2015 to 18 May 2015 (W and SP, 2015)	PM10, PM2.5^b^; daily, 24 h, noon to noon, LVS (38,3 L/min). PM2.5 collected on Quartz/Teflon filters (99).	Beta-ray absorption
Sofia, Bulgaria (SOF)	*National Institute of Meteorology and Hydrology NIMH* (42°39′19.08″ N; 23°23′4.92″ E). Urban background (UB); residential area in the Southeastside of SOF	06–25 Feb 2012, 02–26 July 2012, 22–31 Oct 2012 (W, SU, F 2012); 17 Dec 2012 to 11 Feb 2013; 01–23 July 2013 (W 2012–13, SU 2013)	PM10^b^; daily, 24 h, 8,30 a.m. to 8,30 a.m. LST, (38,3 L/min). PM10 collected on Quartz filters (109).	Gravimetric

a (N): number of collected PM filters.

b PM size fraction considered in the source apportionment study.

### 2.2 PM chemical determinations

The sample collection and chemical analysis at each monitoring site were carried by local laboratories. PM samples were chemically analysed following validated and widely used analytical procedures available in the literature. The resulting input datasets met quality standards in terms of number of samples and robustness of the analytical methods in line with the EU guidelines for source apportionment (Belis et al., 2014). The laboratory procedures and analytical techniques applied for chemical characterisation of PM composition at each site are described below. A detailed list of the species used at each monitoring site is given in Table 2.

**Table 2:** Averaged concentration, and standard deviation (SD), of PM and chemical species (EC/OC, elements, ions, PAHs and LEVO) in Zagreb (ZGR), Budapest (BDP) and Sofia (SOF) used for PMF runs. Chemical concentrations refer to PM_2.5_ samples for ZGR and BDP, and to PM_10_ samples for SOF.

Site			Zagreb (ZGR)		Budapest (BDP)		Sofia (SOF)	
Period			2013 (1 Jan–31 Dec)		2015 (09 Feb–18 May)		2012–13 (06–25 Feb 12, 02–26 July 12, 22–31 Oct 12, 17 Dec 12–11 Feb 13,01–23 July 13)	
			Average	SD	Average	SD	Average	SD
	PM10	μg m^− 3^	27.7	20.2	29.9	15.4	42.2	39.7^a^

	PM2.5	μg m^− 3^	21.9	18.2^a^	17.4	11.1^a^	_	
PM1	μg m^− 3^	16.2	14.3			_	

OC/EC	OC	μg m^− 3^	5.5	5.0^a^	4.3	2.4^a^	_	
	EC	μg m^− 3^	0.7	0.4^a^	0.6	0.3^a^	_	

Ions	SO_4_^2 −^	μg m^− 3^	3.0	2.5^a^	2.0	1.7^a^	5.1	3.7^a^
	NO_3_^−^	μg m^− 3^	2.1	2.4^a^	1.9	2.1^a^	2.0	3.6^a^
	Cl^−^	μg m^− 3^	0.1	0.1^a^	_		0.3	0.7^a^
	NH_4_^+^	μg m^− 3^	_		1.2	1.2^a^	1.7	1.9^a^
	Na^+^	μg m^− 3^	_		0.13	0.11	0.4	0.3^a^
	K^+^	μg m^− 3^	_		_		0.3	0.4^a^
	Ca^2 +^	μg m^− 3^	_		_		0.7	0.5
	Mg^2 +^	μg m^− 3^	_		0.02	0.01	0.07	0.05^a^
Elements	Ag	ng m^− 3^	20.4	2.5	_		8.5	14.0
	Al	ng m^− 3^	_		26.2	23.7	_	
	As	ng m^− 3^	_		_		5.5	14.3
	Ba	ng m^− 3^	52.4	16.2	4.0	2.1^a^	5.6	8.5^a^
	Br	ng m^− 3^	2.0	1.4^a^	2.3	1.1	11.0	21.0
	Ca	ng m^− 3^	77.8	65.2^a^	84.7	90.8^a^	561.8	424.3^a^
	Cd	ng m^− 3^	_		_		2.1	3.4
	Cl	ng m^− 3^	_		28.5	48.2^a^	158.8	304.2
	Co	ng m^− 3^	0.6	0.5	2.2	1.5	_	
	Cr	ng m^− 3^	2.5	1.2	5.4	3.5^a^	3.3	5.0
	Cs	ng m^− 3^	10.0	4.4	_		_	
	Cu	ng m^− 3^	4,4	4.5^a^	1.8	1.5^a^	23.4	29.3^a^
	I	ng m^− 3^	_		_		2.1	4.2
	K	ng m^− 3^	98.5	110.6^a^	152.6	136.4^a^	233.4	183.9
	Fe	ng m^− 3^	58.6	54.1^a^	86.2	61.8^a^	476.2	307.9^a^
	Ge	ng m^− 3^	1.1	0.7	_		_	
	La	ng m^− 3^	14.0	7.1	_		_	
	Mn	ng m^− 3^	2.4	1.7^a^	1.7	1.4^a^	7.3	7.3^a^
	Ni	ng m^− 3^	2.3	1.9	0.7	0.5^a^	2.9	7.3
	P	ng m^− 3^	_		_		265.9	360.6
	Pb	ng m^− 3^	7.1	6.9^a^	8.1	6.2^a^	15.9	33.8^a^
	Rb	ng m^− 3^	0.6	0.7^a^	0.9	0.6	4.8	6.0
	S	ng m^− 3^	283.3	208.4	570.8	446.7	788.0	606.6
	Sb	ng m^− 3^	_		_		5.1	4.7
	Sc	ng m^− 3^	3.0	0.9	_		_	
	Se	ng m^− 3^	_		1.0	0.7^a^	_	
	Si	ng m^− 3^	_		82.4	77.3^a^	_	
	Sn	ng m^− 3^	_		_		4.2	5.8
	Sr	ng m^− 3^	2.6	2.8^a^	1.3	1.0	5.5	11.1
	Ti	ng m^− 3^	2.6	3.8^a^	3.8	3.0^a^	31.0	32.7^a^
	V	ng m^− 3^	0.3	0.8^a^	1.0	0.5^a^	3.9	4.5^a^
	Zn	ng m^− 3^	12.9	10.8^a^	15.0	13.9^a^	55.3	77.8^a^
	Zr	ng m^− 3^	_		_		2.4	3.8
PAHs	BaP	ng m^− 3^	1.9	3.5^a^	_		_	
	BhiP	ng m^− 3^	3.4	5.4^a^	_		_	
	IcdP	ng m^− 3^	1.2	1.8^a^	_		_	
	LEVO	μg m^− 3^	_		0.2	0.2^a^	_	

a Chemical variables used in PMF analysis.

OC/EC content in PM samples (quartz filters) was quantified by means of thermal optical transmittance (TOT) method following the NIOSH-like protocol called Quartz in ZGR (Godec et al., 2016), and the NIOSH 870 protocol in BDP (Maenhaut and Clayes, 2007, Piazzalunga et al., 2011). OC and EC missing data in PM_2.5_ of ZGR (ca. 20%) were reconstructed by regression analysis (R^2^ = 0.95) from OC concentrations in PM_10_ in the same site and the same days (Fig. S1). On the other hand, EC missing data in PM_2.5_ samples were derived by regression analysis from light absorption coefficient α determined on the same site and same samples (R^2^ = 0.63). For SOF, OC/EC data were not available.

Major anions were analysed by ion chromatography in ZGR (Čačković et al., 2009), BDP and SOF. In BDP, water soluble cations were analysed by atomic absorption spectroscopy (AAS), and quantification of ammonium by photometry with indophenol reaction (Kugler et al., 2014, Szigeti et al., 2013). For SOF, cations were also analysed by ion chromatography, following the recommendations and guidelines of EMEP (EMEP, 2001) and WMO/GAW manuals (WMO/GAW, 2016).

Elemental concentrations in ZGR, were measured by ED-XRF (energy dispersive-X-ray diffraction) technique for 22 elements in PM_2.5_ (IAEA, 2013, EPA, 1999). In BDP, elemental concentrations were measured by PIXE for 16 elements (Furu et al., 2015), and by ED-XRF for 5 trace elements (Co, Cr, Rb, Se, Sr) (Osán et al., 2001, Samek et al., 2017) in PM_2.5_ samples. For SOF, the analysis of elements was performed using ED-XRF (Veleva et al., 2014) for the quantification of 25 elements in PM_10_ samples.

Molecular markers are organic species with a high degree of source specificity. Notwithstanding they may undergo transformation processes because of their atmospheric reactivity, some of them such as heavy PAHs and LEVO provide valuable evidence for source characterisation and their use is widely accepted in receptor modelling (Van Drooge and Pérez Ballesta, 2010, El Haddad et al., 2011, Ma et al., 2011, Perrone et al., 2012, Masiol et al., 2012, Khairy and Lohmann, 2013, Jang et al., 2013, Belis et al., 2014, Waked et al., 2014, Vicente et al., 2015, Tan et al., 2017) provided their chemical properties are duly taken into account in the data interpretation. PAHs are markers of incomplete combustion, and they are strongly associated with biomass burning and/or traffic sources at urban sites (Ravindra et al., 2008, Belis et al., 2011). For ZGR, polycyclic aromatic hydrocarbons (PAHs) levels were analysed by HPLC-FLD (high pressure liquid chromatography coupled with a fluorescence detector) (Jakovljević et al., 2015). Three PAHs were used in the data analysis: benzo[*a*]pyrene (BaP), benzo[*g*,*h*,*i*]perylene (BghiP) and indeno[*c*,*d*]pyrene (IcdP). Levoglucosan (LEVO) is a marker for primary biomass burning emissions (Caseiro et al., 2009). In BDP, LEVO was analysed by GC–MS (gas chromatography coupled with mass spectrometry) in PM_2.5_ samples (Gelencsér et al., 2007).

### 2.3 Source apportionment by PMF

Source contributions to ambient PM samples were estimated using PMF (Positive Matrix Factorization), an advanced factor analysis technique based on the work of Paatero and Tapper (1994). PMF uses error estimates to weight data values and imposes non-negativity constraints in the factor computational process. The algorithm accomplishes a weighted least squares fit with the objective of minimizing Q, a function of the residuals weighted by the uncertainties of the species concentrations in the data matrix. The factor model PMF can be written as *X* *=* *GF* *+* *E*, where *X* is the known *n* *×* *m* matrix of the *m* measured chemical species in *n* samples. *G* is an n *×* *p* matrix of factor (source) contribution in every sample (time series). *F* is a *p* *×* *m* matrix of factor compositions (factor profiles). G and F are factor matrices to be determined and *E* is defined as a residual matrix, i.e. the difference between the measurement *X* and the model *Y* *=* *GF* as a function of *G* and *F*.

In this study, the free software US-EPA PMF 5.0 (Norris and Duvall, 2014), implementing the ME-2 algorithm developed by Paatero (1999), was used.

#### 2.3.1 Input data and settings in PMF analysis

The input data pre-processing and the settings used in the PMF runs followed the EU protocol for receptor models (Belis et al., 2014) and are summarised in Table 3. The chemical composition of PM_2.5_ samples was used as input data in PMF analysis for ZGR and BDP, while PM_10_ samples were used for SOF.

**Table 3:** Summary of input data, EPA PMF settings and error estimation (EE) for all the datasets: ZGR-COLD (Zagreb, cold season), ZGR-WARM (Zagreb, warm season), BDP (Budapest) and SOF (Sofia).

	Parameters	Zagreb		Budapest	Sofia
	Dataset	ZGR-COLD	ZGR-WARM	BDP	SOF
Input data and EPA PMF settings	Data type: PM, period	PM2.	PM2.5	PM2.5	PM10
		-cold season 2013	-warm season 2013	Feb-May 2015	-different seasons 2012–13
	No. of samples, *m* (% of tot samples)	174 (96%)	161 (88%)	94 (95%)	99 (91%)
	Species list	SO_4_^2 −^, NO_3_^−^, Cl^−^, Br, Ca, Cu, K, Fe, Mn, Pb, Rb, Sr, Ti, V, Zn, OC, EC, benzo[*a*]pyrene (BaP), benzo[*g*,*h*,*i*]perylene (BghiP) and indeno[*c*,*d*]pyrene (IcdP)		SO_4_^2 −^, NO_3_^−^, NH_4_^+^, Ba, Ca, Cl, Cr, Cu, K, Fe, Mn, Ni, Pb, Se, Si, Ti, V, Zn, OC, EC, LEVO	SO_4_^2 −^, NO_3_^−^, Cl^−^, NH_4_^+^, Na^+^, K^+^, Ca^2 +^, Mg^2 +^, Ag, Ba, Cu, Fe, Mn, Pb, Ti, V, Zn
	No. of used species	20	17	21	17
	PM total mass	Included (TOT VARIABLE-weak)		NOT included in the PMF	Included (TOT VARIABLE-weak)
	Treatment of missing data	YES. EC and OC missing conc since 01th Jan to 14th March 2013 estimated: EC_PM2.5_ by α abs; OC_PM2.5_ by OC_PM10_)	Missing data NOT included	Any missing data (treatment not necessary)	YES. S, Cl, K, Ca, Ti missing 5 days in summer 2012 replaced with median. 8 samples with missing ions excluded
	Treatment of data. ≤ 0 an/or < DL	YES: conc. ≤ 0 replaced by DL/2	YES: conc. ≤ 0 replaced by DL/2	YES: conc. ≤ 0 replaced by DL/2	YES: conc. ≤ 0 replaced by DL/2
	Uncertainty (u)	Analytical uncertainty: + 10% for all species	Analytical uncertainty. + 10% for all species	Anal. uncertainty: + 10% for all species	Analytical uncertainty: + 10% for all species
	Extra modelling u (%)	0	0	0	10
	N factors, *p*	4 to 6 (5 as final solution)	4 to 6 (5 as final solution)	4 to 6 (5 as final solution)	5 to 7 (6 as final solution)
	Lower limit for norm. factor contrib g_*ik*_^a^	− 0.2	− 0.2	− 0.2	− 0.2
	Robust mode	Yes	Yes	Yes	Yes
	Seed value	Random	Random	Random	Random
Rotational tools	FPEAK test	YES (Fpeak − 0.5; dQ_rob_ 1.93%)	YES (Fpeak 0)	YES (Fpeak 0)	YES (Fpeak 0)
	Constrain	None	None	YES. LEVO pull down max Fact 1-SEC, 4-SOIL, 5-TR; dQ_rob_ 0.91%	YES. SO_4_^=^ pull down max Fact 2-TR; dQ_rob_ 0.50%
Error estimation	N bootstraps in BS	100	100	100	100
	R2 for BS (PMF default value)	0.6	0.6	0.6	0.6
	BS block size (suggested value)	23	15	8	6
	DISP dQ_*max*_	4,8,15,25	4,8,15,25	4,8,15,25	4,8,15,25
	DISP active species	All “not weak”	All “not weak”	All “not weak”	All “not weak”

a IN EPA PMF, the lower limit of the normalised contributions is set − 0,2, since allowing a small negative value helps PMF accept true rotations even in the presence of a large number of zero values in some G factors.

Considering the abundance of samples, the ZGR dataset was divided into two subsets, one including the cold months (COLD: Jan-March and Oct-Dec) and the other including the warm months (WARM: March–August). PMF analyses were run for the entire dataset (not shown) and separately for each subset in order to minimise the influence of possible seasonal variations in factor profiles. For BDP and SOF the standard procedure was followed because there were not enough samples in the datasets for split runs.

The number of PM samples (*n* raw samples) processed in each PMF analysis ranged between 94 and 174, with a % of modelled samples in the range of 88% - 96%. Few samples were excluded for the PMF analysis: those with many missing values or those influenced by exceptional circumstances (e.g. New Year's Eve fireworks).

The number of species actually used for the runs ranged between 17 and 21. Species were classified as “weak” when: the number of samples below the detection limit was > 50%, species were bad modelled, or species are subjected to degradation (e.g. PAHs).

In PMF analysis, uncertainty estimation is particularly critical because every entry is weighted according to its uncertainty, and input uncertainty in PMF should account for all the uncertainty components that contribute to residuals. The uncertainty used for the PMF analysis was calculated according to Norris and Duvall (2014). In order to account for unknown sources of uncertainty, the analytical uncertainty provided in the original dataset (Table S1) was incremented by 10% for all species.

A first estimation of the number of factors *p* was accomplished by analysing step-wise the Q values of multiple runs with increasing number of factors. The quality of the fit (scaled residuals) and the interpretability of the results (in terms of chemical profile and time trend) was evaluated to identify possible factors representing more than one source category or sources split over more factors. The *p* was then refined using the techniques described in the following sections.

#### 2.3.2 Optimisation of model solution

Even when a minimum in the least squares fitting process is found, the solutions obtained in factor analysis are not unique. Because of the free rotation of matrices there is a family of solutions that are equally fit; so called rotational ambiguity (Paatero et al., 2002). In this work, the rotational ambiguity of the PMF solutions was explored with the FPEAK tool for multiple values of the parameter (ranging between − 1 and + 1). The impact of small rotations on the Q values, F and G matrices as well as scaled residuals was negligible in the majority of the cases. FPEAK value ≠ 0 was chosen only for ZGR-COLD (FPEAK = − 0.5; dQ_rob_ 1.93%) to sharpen the G matrix.

In advanced PMF, known source chemical profiles or contributions can be used to constrain a model run to improve the physical relevance of factors (Sofowote et al., 2015, Amato and Hopke, 2012). Considering that LEVO is a unique tracer for biomass burning source, in BDP a constrained solution was selected with LEVO pulled down maximally (“soft pulling”, dQ = 0.91%) in factors where the contribution of biomass burning was meaningless. Similarly, in SOF traffic was constrained with sulphates pulled down maximally (“soft pulling”, dQ = 0.50%) in order to match the range of relative mass observed for that compound in the traffic chemical profiles of SPECIEUROPE (Pernigotti et al., 2016).

#### 2.3.3 Error estimation and diagnostic of PMF solutions

Error estimation (EE) methods included in the US-EPA PMF 5.0 software were used for analysing factor analytical solutions (Norris and Duvall, 2014). These EE methods capture the uncertainty of PMF analyses due to both random errors and rotational ambiguity.

Bootstrap (BS) is most useful to detect and estimate possible random errors due to disproportionate effects of a small set of observations on the solution. In this study, BS were used for quantifying the uncertainty of a solution, and for identifying factors that have a low degree of reproducibility (Brown et al., 2015). One hundred BS runs were performed for each dataset to ensure the robustness of the statistics, and the 5th and 95th percentiles were considered as the BS uncertainty range for each factor profile.

Displacement (DISP) was used to explore the rotational ambiguity in the solutions more explicitly by assessing the largest range of source profile values without an appreciable increase in the Q-value. In this technique, each fitted element (only “strong” species) in a factor profile is “displaced” from its fitted value far enough so that Q increases by a predetermined amount called dQ_*max*_. For each dQ_*max*_, DISP is executed, and upper and lower interval estimates of the perturbed variable yield an uncertainty estimate for each species in each factor profile. In DISP the focus is on how often factors change enough to exchange identities (swap), indicating a not-well defined solution (Paatero et al., 2014). If more than a few swaps occur for the smallest dQ_*max*_, there are either too many factors or the rotational ambiguity is significant. On the contrary, if no or only a few swaps occur, the solution is to be considered acceptable from a statistical point of view.

#### 2.3.4 Factor attribution to sources

In this study, the names attributed to the sources are coherent with the EC-JRC SPECIEUROPE (Pernigotti et al., 2016) nomenclature. The attribution of factors to source categories was supported by quantitative comparisons of the factor chemical profiles (μg μg^− 1^) with PM profiles measured at the source and profiles from other source apportionment studies available in the literature. To that end, the Pearson coefficient, expressed as Pearson distance (*PD = 1* − *r*), and the standardised identity distance (SID, Belis et al., 2015) were used to calculate the similarity between the factors and the reference source profiles available in the public datasets: EC-JRC SPECIEUROPE and US-EPA SPECIATE (Simon et al., 2010). The task was accomplished using the DeltaSA tool (http://source-apportionment.jrc.ec.europa.eu/). Explained variation values (EV) for species and factor contributions time series were also inspected in order to check seasonal patterns.

### 2.4 The geographical origin of pollution sources

As a preliminary assessment of whether the measured pollutants were emitted locally or not, the wind speed and direction measured at the receptor site and the PMF results were analysed by means of the conditional probability function (CPF) technique (Ashbaugh et al., 1985) as implemented in the Openair software R tools (Carslaw and Ropkins, 2012). The CPF provides a preliminary indication of the quadrant and distance of the source. Sources were considered local when the maximum probability was located at the Cartesian origin (wind speed < 2 m/s). However, local winds could be influenced by local factors (e.g. orography, buildings) and advected secondary pollutants originated in distant locations may reside at the monitoring site under low wind conditions. Therefore, the classification of the sources based on CPF results was further refined by comparing the analysis of backward trajectories, which provides information about the geographical origin of pollutants on a wider scale, with mesoscale emission maps of the ECLIPSE EMISSIONS DATASET (http://eclipse.nilu.no/) for the reference years 2010 (Fig. S5) and 2015. The trajectories of air masses during the sampling periods were calculated with the Flexible Particle Dispersion Model (FLEXPART). The potential contribution source function (PSCF, Ashbaugh et al., 1985) was then used to identify the origin over large geographical scale of the PM sources identified with PMF and associated gaseous pollutants/precursors.

#### 2.4.1 The Flexible Particle Dispersion Model (FLEXPART)

The Flexible Particle Dispersion Model (FLEXPART) was used to acquire residence time (sensitivity) plumes (Stohl et al., 1998, Stohl and Thomson, 1999, Stohl et al., 2005, Seibert and Frank, 2004). FLEXPART runs account for grid scale wind as well as for turbulent and mesoscale wind fluctuations. Drift correction, to prevent accumulation of the released computational particles, and density correction, to account for the decrease of air density with height, were both applied. Five and ten-day backward runs with release of 20,000 air parcels every hour at 300 m above ground level were produced. Residence times above 3000 m were excluded as their influence on surface air mass was considered negligible.

#### 2.4.2 Potential source contribution function (PSCF)

The probability of a trajectory circulating over a cell (1° × 1°) reaching the receptor site when the contribution of each source is above the 90th percentile was calculated by PSCF analysis (Eleftheriadis et al., 2009) on the basis of FLEXPART runs. To down-weight cells with few trajectory endpoints, the number of endpoints in each cell was compared with the total number of endpoints assuming a binomial distribution and implementing a binomial filter (see Supplemental S6). Coefficients 0.1, 0.3, 0.5 and 0.7 were used for cells with probabilities < 1%, 1–5%, 5–10% and 10–15%, respectively. In general, five-days backward trajectories runs have been used in this study. However, longer trajectories (up to 10-days) runs provided more interpretable results for the PM fraction deriving from re-suspension of crustal material likely due to its higher residence times in atmosphere that led to longer transported distances. More details on the PSCF analysis are given in the Supplementary Material (S6. Details on the PSCF analysis).

## 3 Results and discussion

### 3.1 PM mass concentration and chemical composition

In Table 2 are shown the average PM concentrations and chemical species used for PMF runs in the three study sites. The highest PM_10_ concentrations were measured in SOF, in line with the increasing N-S gradient reported by Putaud et al. (2010) for urban background (UB) PM_10_ levels in Europe. The percentage of exceedances of the daily PM_10_ limit value (50 μg m^− 3^) during the period of study was 13% for ZGR, 12% for BDP and 21% in SOF. The PM_2.5_/PM_10_ ratio ranges between 0.74 in ZGR and 0.55 in BDP and fall within the range observed in European UB sites.

The EC contribution to PM_2.5_ in ZGR and BDP (4–5%) fall in the lower range of urban background values observed in Central Europe (13%) and Southern Europe (8%) (Putaud et al., 2010). On the other hand, relatively large OC values lead to high OC/EC ratios in ZGR (8,7 ± 4,8) and BDP (7,4 ± 2,5). Such a carbonaceous composition is consistent with a considerable contribution of secondary organic aerosol (Pio et al., 2011).

Sulphate accounted on average for 7% (BDP), 11% (ZGR) and 12% (SOF) of PM concentrations while nitrate for 5% (SOF), 6% (BDP) and 8% (ZGR). Such shares of major inorganic ions in the PM composition are comparable with those reported for urban sites across Europe (Putaud et al., 2010).

The levels of PM and the chemical composition of the studied datasets are in line with the values reported for urban background sites in this area of Europe confirming their suitability to be used for the estimation of the average source contributions in their respective cities.

### 3.2 PMF results: factors and source attribution

**Fig. 2:**
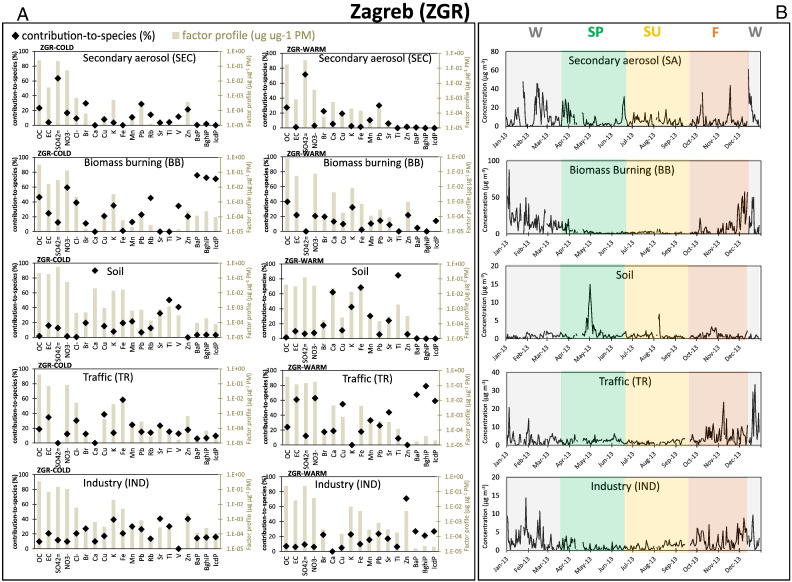
Factor contribution-to-species (%; the share of each species apportioned to the factor) and chemical factor profiles (μg μg PM^− 1^; the relative mass contribution of each species to PM mass of the factor) (left panel, A), together with temporal evolutions (μg m^− 3^) (right panel, B; W = winter (grey), SP = spring (green), SU = summer (yellow), F = fall (red)), for the five factors/sources identified by PMF in Zagreb (PM_2.5_ data).

**Fig. 3:**
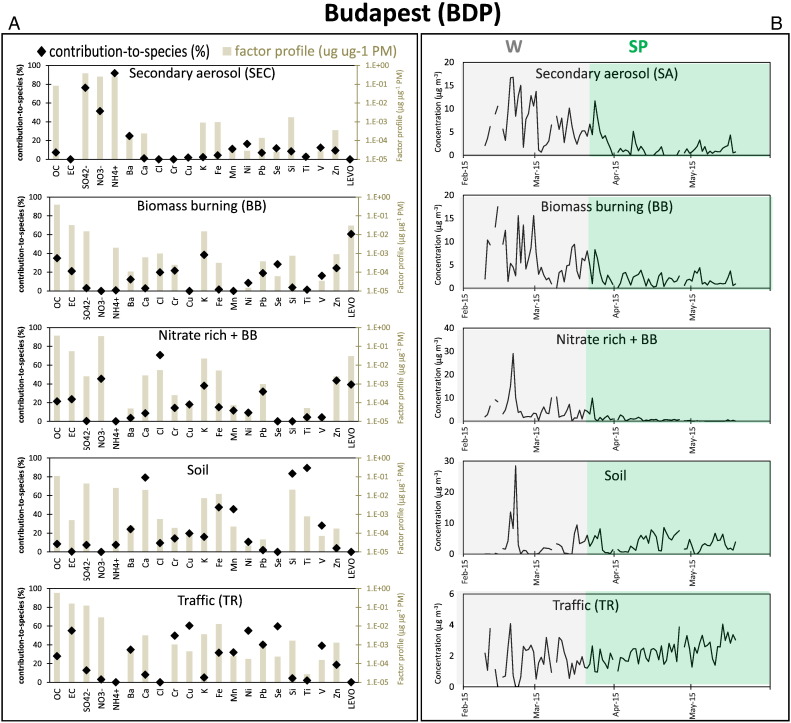
Factor contribution-to-species (%; the share of each species apportioned to the factor) and chemical factor profiles (μg μg PM^− 1^; the relative mass contribution of each species to PM mass of the factor) (left panel, A), together with temporal evolutions (μg m^− 3^) (right panel, B; W = winter (grey), SP = spring (green)), for the five factors/sources identified by PMF in Budapest (PM_2.5_ data).

**Fig. 4:**
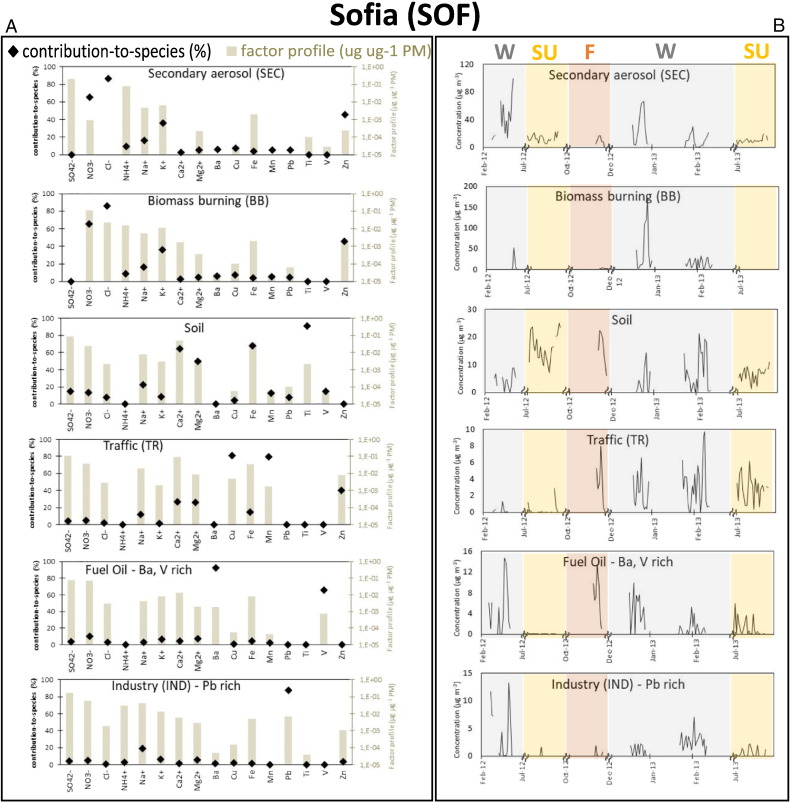
Factor contribution-to-species (%; the share of each species apportioned to the factor) and chemical factor profiles (μg μg PM^− 1^; the relative mass contribution of each species to PM mass of the factor) (left panel, A), together with temporal evolutions (μg m^− 3^) (right panel, B; W = winter (grey), SU = summer (yellow), F = fall (red)), for the five factors/sources identified by PMF in Sofia (PM_10_ data).

The most physically plausible PMF results were obtained with five-factor solutions in ZGR (both COLD and WARM) and BDP, and with a six-factor solution in SOF. The factor contribution-to-species (% of each species apportioned to the factors), chemical factor profiles (expressed as the relative mass contribution of each chemical species to PM mass, μg μg^− 1^) and factor time-series (μg m^− 3^) are given in Fig. 2, Fig. 3, Fig. 4. The chemical profiles of the solutions' factors corresponding to primary sources passed the DeltaSA similarity tests with the corresponding reference source profiles for at least one of the tested distances (PD and SID).

#### 3.2.1 Zagreb

PMF results and EE diagnostics for the final five-factor solution for ZGR PM_2.5_ (Fig. 2A) indicate that results are stable and that all species are well predicted, with low Q/Q_exp_ values (< 3) except for Mn and NO_3_^−^ (Table S2). No swaps occurred in DISP, indicating that the solution is well defined. The number of correctly mapped factors in BS was high (> 77%) for all factors. No significant differences in the source allocation was observed when comparing summer and winter PMF runs with a single PMF run for all the samples.

**Secondary aerosol (SEC)** is a factor characterised by the highest share of sulphate in ZGR during all seasons (Fig. 2A). In addition, OC and anthropogenic elements (e.g. Br, Pb) indicate aged primary emissions mixed with secondary sulphate and OC.

**Biomass burning (BB)** factor, presents high concentrations of typical markers: OC, Cl^−^, K, Rb, and PAHs. Seasonal variation of this factor (Fig. 2B) is coherent with domestic heating during cold months. BB is the highest contributor to OC around the year and of PAHs during the cold season.

The **SOIL** factor is characterised by high concentrations of typical crustal components, such as Ca, K, Fe, and Ti, (Belis et al., 2013). The **Traffic (TR)** factor presents the highest share of EC and Cu concentrations, two markers associated with vehicle emissions (Viana et al., 2008). This factor is the highest contributor to NO_3_^−^ around the year and to PAHs during the warm season.

A factor, which is the main contributor of Zn, and important contributor of K, Pb and PAHs, was attributed to metal production industry **(IND)** source.

#### 3.2.2 Budapest

Diagnostics indicate that all PM_2.5_ species are well predicted (Q/Q_exp_ < 3) also at this site and no swaps were observed in the DISP test. The factors were correctly mapped in the BS test at least 87% of the time (Table S2).

The **secondary source SEC** is the main contributor of inorganic ions (ammonium sulphate and nitrate) and includes a considerable level of organics (Fig. 3A).

The **BB** source is the one with the highest levoglucosan shares (61%), a tracer of biomass burning emissions (Simoneit, 2002, Cordell et al., 2016), and is also characterised by high levels of K and OC.

Another factor characterised by nitrate, OC, EC, K and Cl with a considerable concentration of levoglucosan (39% of this species) was attributed to either secondary aerosol deriving from primary BB or aged aerosol mixed up with fresh BB emissions. The chemical profile of this factor, named **Nitrate rich + BB**, suggests a complex history. It presents a strong seasonal trend with highest concentrations during winter (Fig. 3B) that may be attributed to both the highest contribution from biomass burning for residential heating during the coldest months and the highest partitioning in the same period of nitrate in the solid phase favoured by low temperature and high relative humidity conditions. The lower content of levoglucosan (in particular the ratio levo/K) in Nitrate rich + BB is coherent with the hypothesis of a higher degradation due to a longer transport (see Section 3.4.2). However, the most distinct features for these factors are their time trends. The BB presents a rather typical seasonal trend for this source with higher contributions in winter that gradually decrease to achieve minimum levels in late spring. On the other hand, the Nitrate rich + BB factor is characterised by a distinct episode in the second half of February followed by minor isolated events in March. A more detailed discussion on these two factors is provided in the Supplementary Material (S4. PMF analysis).

Also in this site the **SOIL** factor is identified by the dominance of crustal elements, such as Ca, Fe, Mn, Si and Ti. The source attribution is supported by the high correlation (*R* = 0.89) between the time series of this factor and mineral dust estimated using empirical coefficients (Putaud et al., 2010).

The traffic (**TR**) source is identified by the presence of OC and EC as main components deriving from vehicle exhaust accompanied by indicators of non-exhaust traffic particles (e.g. Ba, Cu, Fe) (Amato et al., 2010).

#### 3.2.3 Sofia

The diagnostic tests indicate suitable Q/Q_exp_ values for the majority of the elements with the exception of Ti, V, Mn, Cu, Cl^−^ and NO_3_^−^ (Table S2). No swaps were observed in the DISP test, and in the BS test results factors were mapped in ≥ 99% of the runs with the exception of the IND Pb rich factor that was mapped 89% of the time. The higher uncertainty of this factor may be associated to the presence of peak events in winter 2012 (Manousakas et al., 2017).

As with the previous sites, the Secondary (**SEC**) factor is dominated by inorganic ions (ammonium sulphate) like in the two other sites (Fig. 4A).

The biomass burning (**BB**) factor contains high concentrations of NO_3_^−^, Cl^−^, K^+^ and Zn. As in the previous sites, high levels of NO_3_^−^ are associated with BB source indicating a certain degree of ageing.

As in the previous sites, the **SOIL** factor is dominated by crustal elements.

The traffic (**TR**) factor is noticed by the presence of NO_3_^−^ deriving from the ageing of exhaust gases and by species that are markers for non-exhaust traffic emissions such as Cu, Mn and Zn, together with Fe, Ca^2 +^ and Mg^2 +^ (see 3.2.1, 3.2.2). In particular Zn is used as an additive in lubricant oils for vehicles (Salvador et al., 2007) and is associated with tyre wear (Pant and Harrison, 2013). The quantification of the exhaust component in this factor may have been underestimated due to the lack of EC and OC data for this site.

The **Fuel Oil** factor is characterised by high concentrations of Ba and V, key tracers for fossil fuel/heavy residual oil combustion processes (Moreno et al., 2010). The chemical profile of this factor has affinities with power plants and coal combustion.

The factor **IND** is characterised by high levels of Pb (accounts for 90% of this element), a marker of industrial emissions (Taiwo et al., 2014), which has been associated with local glass production and processing of aluminium and copper alloys (Bulgarian National Emission Register). IND is a rather intermittent source that reached high concentrations only during winter 2012 (Fig. 4B).

### 3.3 PM sources under a regional (Danube) and European perspective

**Fig. 5:**
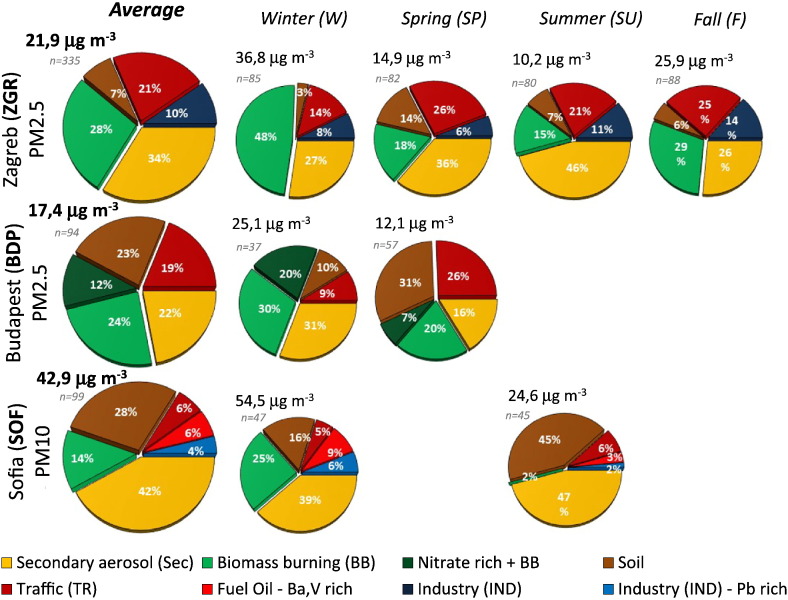
Average and seasonal source contribution (cumulative percentage) to the PM by the PMF model in the three cities of the Danube macro-region.

Source contribution estimates (SCEs) to the PM mass concentration on annual and seasonal bases at the three sites are presented as pie charts in Fig. 5 and in mass concentration (μg m^− 3^) in Table S3.

Four sources were found to be common between the sites and their sum represents > 85% of the PM mass: **SEC**, **BB**, **SOIL** and **TR**.

The **SEC** source observed in this study is dominated at all sites by sulphate rich aerosol with a variable content of nitrates (factor profiles, μg μg^− 1^ PM, in Figs. 2A to 4A). The **SEC** source was found to be the major source in SOF (42%) and ZGR (34%) and among the three most contributing sources in BDP (22%) (Fig. 5). In European urban areas, the median relative contribution from secondary sources (mainly ammonium sulphate and nitrate) is 40% (± 11%) of PM_2.5_ (Belis et al., 2013), which is slightly higher than our results for ZGR and BDP. On the other hand, the median contribution of **SEC** to PM_10_ observed in SOF (42%; 13 μg/m^3^), fall in the highest quartile of those reported for Europe where the median SIA contribution to urban PM_10_ is 10 μg/m^3^ corresponding to 30 ± 6% (Belis et al., 2013). These results highlight the high impact of sulphur dioxide sources in SOF as a consequence of advection processes (see Section 3.4.3).

Biomass burning is the largest source category in BDP with two factors closely linked to it (**BB** and **Nitrate-rich** **+** **BB**) which together add up to 36% of PM_2.5_. Moreover, it is also the second largest source in ZGR (28%) and the third in SOF (14%). Considering that, in Europe, **BB** represents on average 14 ± 6% of PM (Belis et al., 2013), the contributions observed in ZGR and BDP are notable. As expected, the maximum contributions are observed in the cold season when they reach 48% in ZGR, 30% in BDP (50% if **BB** and **Nitrate rich** **+** **BB** are pooled) and 25% in SOF. The observed contributions of **BB** in summer are usually associated with forest/vegetation fires.

**SOIL** is the second largest source in BDP (23%) and SOF (29%) while it represents the smallest contribution in ZGR (7%). In SOF and ZGR the contributions from this source are in line with other European cities, on average 24 ± 11% for PM_10_ and 9 ± 8% for PM_2.5_ (Belis et al., 2013) while the contribution in BDP (PM_2.5_) is relatively high. The **SOIL** time series observed in this study shows high contribution episodes that are associated with long-range transport of desert dust (Section 3.4).

In this study **TR** includes the exhaust emissions and the re-suspension of road dust which is a mixture of particles deriving from vehicle wearing (e.g. tyres, brakes) and soil deposited on the pavement. In the studied sites, **TR** contributes on average to one fifth of PM_2.5_ which is line with contributions typically reported for European cities for this size fraction (21 ± 12%) (Belis et al., 2013). On the other hand, the relatively low **TR** contribution to PM_10_ in SOF (6%) is in part attributable to the lack of the carbonaceous fraction, which is a typical component of vehicle exhaust emissions, in the input dataset of this site.

**IND** sources were only identified in ZGR and SOF and account for 10% and 4% of PM respectively. **IND** sources are heterogeneous as are the underlying industrial sites, their operations and the meteorological conditions (e.g. wind direction; Taiwo et al., 2014). As a consequence, their contributions to PM in European urban sites are highly variable, ranging between 2 and 37%. In the case of ZGR the chemical profile is attributable to metal production industry, while in SOF it has been associated with glass production and metal processing plants.

**Fuel Oil** is a source with a small contribution (6%) identified only in SOF which is chemically compatible also with coal combustion. It shows higher levels in winter indicating a probable origin from centralised heating systems or power plants. High level episodes for this source have been associated with long range transport (Section 3.4).

### 3.4 Identification of geographical areas contributing to pollution

In this study CPF graphs (Figs. S2, S3, S4) have only been used to identify the sources that are of predominantly local origin (city and surrounding region) and those which are affected by long-range transport. Nevertheless, the interpretation of CPF is not straightforward. In Sofia, for instance, the wind at the city scale is influenced by the topography of the surrounding valley leading to prevailing winds from W-NW and E-SE and highest westerly wind speeds. To overcome this CPF limitation, the classification in either local or non-local was made considering also the PSCF and the analysis of the emission maps. PSCF analysis applied to local sources generally resulted in non-statistically significant probability maps (because there is no relationship between the trajectories and the source contributions) and their geographical patterns were not coherent with those of the emission maps.

According to this analysis, **TR, IND** and **BB** can be attributed to local or regional sources (with the exception of BB in BDP) while **SEC, SOIL** and **Nitrate rich +** **BB** are from sources further away from the receptor. **Fuel oil** presented an intermediate situation with both a limited contribution from local sources and some long-range transport episodes.

**Fig. 6:**
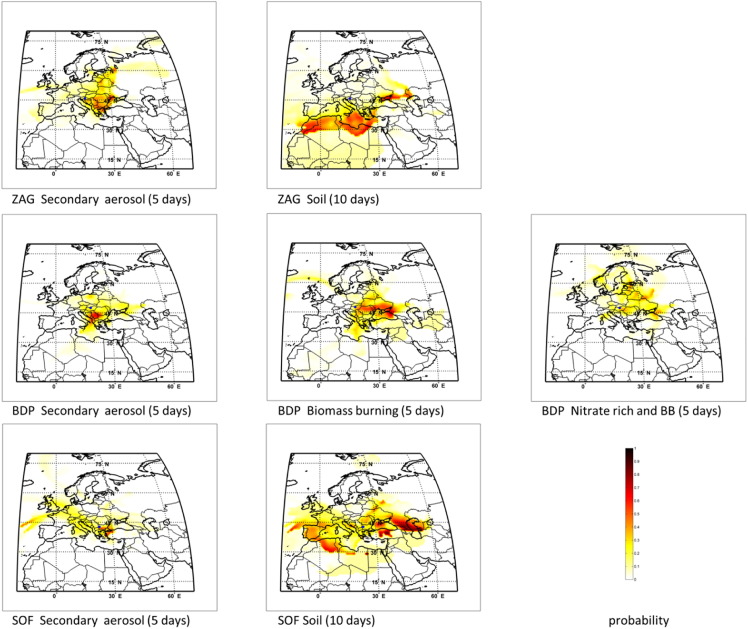
FLEXPART PSCF (only sources affected by long-range transport). ZAG: Zagreb; BDP: Budapest; SOF: Sofia.

The FLEXPART PSCF analysis focused on the group of sources that underwent significant long-range transport and the resulting maps are shown in Fig. 6. PSCF frequency maps indicate the geographic origin of the air masses (transnational) that reach the study site when the contribution of the studied source is the highest. Nevertheless, high PSCF values do not necessarily indicate a source location, especially when dealing with secondary pollutants. To check the correspondence between the trajectories and emissions, the PSCF maps were interpreted by visual comparison with the emission maps.

#### 3.4.1 Zagreb

According to FLEXPART PSCF analysis, the **SEC** source is mainly transported from Eastern and North-Eastern Europe (Fig. 6). The prevailing origin of **SEC** is associated with air masses circulating over the Balkan Peninsula in correspondence with considerable SO_2_ emission sources (Fig. S5). North-eastern trajectories match SO_2_ hotspots in Ukraine, Belarus and the area around St. Petersburg (Russia).

FLEXPART PSCF analysis for the **SOIL** source show two distinct features corresponding with the Saharan area in Northern Africa (mainly Libya and Algeria) and the area of the Caspian Sea corresponding to the North-western corner of the Turkestan desert. High PSCF scores over the Mediterranean Sea are considered only indicative of the transport path and not as a source area.

The **BB** and **TR** sources in ZGR appear to be mainly influenced by local contributions, as indicated by the CPF plot (Fig. S2) with the highest frequencies near the origin (lowest wind speed). CPF frequencies indicate that **TR** source areas increase towards South-East corresponding to the highest traffic density in the city of Zagreb (the receptor site is located to the North-West of downtown). CPF plots show high **IND** source contributions when easterly winds blow, pointing out the influence of the local industrial emissions district located to the east of the city.

#### 3.4.2 Budapest

The **SEC** source FLEXPART PSCF shows high frequencies from the Balkan peninsula that match SO_2_ emission hot spots in Croatia, Serbia, Montenegro, Romania and Bulgaria (Figs. 6 and S5).

The FLEXPART PSCF analysis tracks the origin of **Nitrate rich** **+** **BB** source over Romania, and Ukraine and further east- and northwards to the Baltic states and southern Finland (Helsinki), Warsaw and the areas of Moscow and Krasnodar in Russia. The interpretation of these results is that nitrate-rich aerosols deriving from primary emissions in distant large cities are transported over long distances and enriched with biomass burning emissions located to the east of the monitoring station. In the ECLIPSE emission inventory, areal PM_10_ emissions from the residential sector are reported in Hungary and in Romania (Fig. S5). In addition, during the period of maximum **BB** contribution at this site, a high number of open field fires, likely associated with agricultural practices, were recorded by satellite images in Ukraine and western Russia (Fig. S5).

The CPF analysis for **SOIL** and **TR** sources indicates high frequency corresponding with southern winds at intermediate speed (3 m/s) (Fig. S3). Moreover, no clear long range transport pattern is observed in the PSCF analysis suggesting that the main contribution for these two categories is local. SOIL and TR present different time series, a common origin for these two sources can therefore be excluded.

#### 3.4.3 Sofia

The FLEXPART PSCF analysis identifies significant contributions of **SEC** source from North-western Turkey, including the regions of Istanbul and Izmir, and from southern Bulgaria (Fig. 6), all areas characterised by SO_2_ emission hotspots from stationary sources and maritime traffic.

At this site the episodes with high levels of **SOIL** (conc. > 20 μg/m^3^) are always associated with long range transport of desert dust from a variety of geographical areas (Fig. S6). In summer 2012 two episodes were observed (the first of which on 6th July and a second one on 23rd–26th July) with origins in the area to the East of the Caspian Sea (Kazakhstan, Karakum desert). On 23rd–24th October of the same year, another long range transport of **SOIL** from Karakum desert, Turkey and Arabic Peninsula was observed. In addition, a Saharan dust episode was recorded between 2nd and 5th February 2013. The same episode has been documented by analyses of the EMEP intensive measurement period (Alastuey et al., 2016).

**Fuel oil** is likely deriving from local emissions from district heating systems or power plants. However, two episodes of long range transport have been identified on 18–19/2/2012 and 25/10/2012 where air masses circulated over the northern Balkan peninsula (Serbia and Romania).

There are no indications of long range transport for **BB**, **TR** and **IND** sources, suggesting a dominant contribution from local sources for these categories. This is supported by high frequencies at low wind speed in the CPF analysis (Fig. S4).

## 4 Implications for policy

The number of source categories contributing to PM mass in the studied sites ranged between five and six, four of which: **SEC**, **TR**, **BB** and **SOIL** can be considered typical sources for the Danube macro-region that were identified in all the studied locations. The remaining sources derive from specific point sources (e.g. industrial) or processes (interaction between aged aerosols and fresh emissions).

A prevailing local origin was found for **TR**, **BB**, and **IND**. Industrial sources are: metallurgy in ZGR and both glass production and metal processing in SOF. The only exception is BB in BDP where the contribution from agricultural fires in Eastern Europe adds up to the one from local residential heating. On the other hand, the **SEC** source, which is one of the major sources contributing to PM (22%–41% on average, depending on the site) was often found to be transported from areas beyond the EU borders located either in the Balkan Peninsula or in Eastern Europe. Advection from distant areas is significant also for **SOIL** which highest levels, determined by long-range transport of dust from the Turkestan or the Sahara deserts, reach contributions between 35% and 77% of the PM. The high levels associated with transport of desert dust observed in this study (especially in ZAG and SOF) is coherent with the west-east gradient for this kind of sources reported for the Mediterranean areas of Europe (Diapouli et al., 2017). However, for yearly averages it must be taken into account that long range transport adds up to background levels of SOIL (8% - 30% of PM) deriving from local sources such as re-suspension from agricultural fields and bare soil areas, industrial fugitive dust emissions and unpaved road dust.

The variation between the average contributions of sources over the whole studied period and the ones observed when the levels are above the limit value (days > LV) was used to point out sources that are likely to be responsible for exceedances in the studies areas (Table 4).

**Table 4:** Variations in the contributions (%) of the main sources when concentrations exceed the annual limit values. The change in the relevance of the source when concentrations are above the limit value is indicated between parenthesis (↑: increase, ↓: decrease, ≈: unchanged).

Site	ZGB		BDP		SOF	
Source	Whole period	Days > LV	Whole period	Days > LV	Whole period	Days > LV
Secondary (SEC)	34	35 (≈)	22	23 (≈)	42	38 (≈)
Nitrate/biomass burn.	–	–	12	18 (↑)	–	–
Biomass burning (BB)	28	42 (↑)	24	21(≈)	14	27 (↑)
Soil	7	2 (↓)	23	17 (↓)	28	15 (↓)
Traffic (TR)	21	12 (↓)	19	5 (↓)	6	5 (≈)

The relative contributions of SOIL and TR tend to decrease when the concentrations of PM are high while the relative share of BB (alone or combined with secondary) grows with the PM level. The relative contribution to PM of secondary processes is always sizeable and no significant variations are observed between low and high PM concentrations. On the basis of this analysis, the key sources to tackle in order to keep the concentrations below the limit values are the combustion of biomass, either domestic or agricultural, and the combustion processes responsible for SO_2_, NO_x_ and NH_3_ emissions which are precursors of secondary aerosol formation.

The study outcome suggests that measures to reduce atmospheric pollution in the cities of the Danube macro-region require action at different levels. At the local level, measures to control diffuse sources from domestic heating and traffic are already under consideration in the air quality plans required by the EU Air Quality Directive 2008/50/EU. For diffuse sources, the impact of technological measures (upgrade of vehicle fleet and introduction of efficient stoves) are likely to be insufficient and should, therefore, be accompanied by structural and behavioural changes. More effort is needed to address diffuse emissions from the domestic heating sector where the use of solid fuels in outdated appliances is contributing substantially to PM pollution in this area. The implementation of the European directive on national emission ceilings (NEC) and that on medium combustion plants (MCP) to achieve an abatement of SO_2_ emissions from point sources are important at both national and EU level. In addition, reducing ammonia emissions in the agriculture sector would be an efficient way to abate secondary PM_10_ and PM_2.5_. However, the entity of the long range pollution transport from EU neighbours detected in this study could reduce the effectiveness of the efforts of EU Member States to comply with the limit values. Additional action is, therefore, needed to control transnational emissions in EU neighbour countries with particular reference to the energy production, industrial and shipping sectors. Notwithstanding the indirect impact of agricultural practices on air quality (e.g. open field burning). In this regard, it is essential to involve all the relevant actors and assess possible interactions between sectorial policies. International instruments such as the CLRTAP Convention protocols and the MARPOL Annex VI provide such tools. Also the EU neighbour based policy with the objective of transfering the EU legislation (EU *aquis*) to accession countries could be a powerful tool to progress towards the improvement of the air quality standards within and beyond the EU borders.

## 5 Concluding remarks

In the present work, the causes of PM pollution in three selected urban locations of the Danube macro-region (ZGR, BDP and SOF) were identified using a combination of datasets and modelling tools. The adopted approach that combines source apportionment receptor models (PMF) and analysis of trajectories (PSCF) obtained with Lagrangian models (FLEXPART) led to quantifying the contributions from different sources and documenting their geographical origin.

The comparison of the chemical composition and mass concentrations of the input datasets used for this study with the data from monitoring networks and scientific literature indicated that they are representative of the studied area including the seasonal variations. The robustness of the source contributions estimates is also the result of applying the most up-to-date methodology and quality assurance procedures from widely recognised technical protocols. Moreover, the approach of comparing factors with reference source profiles adopted in this work ensured the traceability of the source nomenclature for comparison with other studies.

On the basis of the present analyses, policy options to tackle the main sources: **SEC**, **TR**, **BB** and **SOIL** in the cities of the Danube macro-region are discussed. However, for a case by case more in-depth analysis at the local scale it would be necessary to replicate the methodology adopted in this study in other urban areas of the macro-region.

## Appendix A Supplementary data

Additional graphs and methodological notes. Image 1
