# Supplementary material for: Sources and geographic origin of particulate matter in urban areas of the Danube macro-region: The cases of Zagreb (Croatia), Budapest (Hungary) and Sofia (Bulgaria)
Source: Sci Total Environ. 2018 Apr 1;619-620:1515–29. doi: 10.1016/j.scitotenv.2017.11.092 (PMC5821697; doi:10.1016/j.scitotenv.2017.11.092)
Supplement: Supplementary file 1 — Additional graphs and methodological notes. [file mmc1.pdf]

**Sources and geographic origin of particulate matter in urban areas of the Danube macro-region: the cases of Zagreb (Croatia), Budapest (Hungary) and Sofia (Bulgaria)**

M.G. Perrone, S. Vratolis, E. Georgieva, S. Török, K. Šega, B. Veleva, J. Osán, I. Bešlić, Z. Kertész, D. Pernigotti, K. Eleftheriadis, C.A. Belis*

*corresponding author: claudio.belis@ec.europa.eu

## Supplementary Material

## S1. Description of the sampling sites

PM samples were collected at three urban background (UB) sites representing different situations within the Danube Region: Zagreb (Croatia) and Sofia (Bulgaria), from the Balkan Peninsula, and Budapest (Hungary), from the Visegard area.

Zagreb – ZGR. The ZGR site (ZGR: 45°50’07.87” N; 15°58’38.86” E) was set up in the Northeastern side of the city of Zagreb (750.000 inhabitants), bordered by hills to the north. The sampling site itself was located at a low hill altitude of 200 m a.s.l. The ZGR site is representative of a typical residential area, quite close to the city center (about 2 km away)

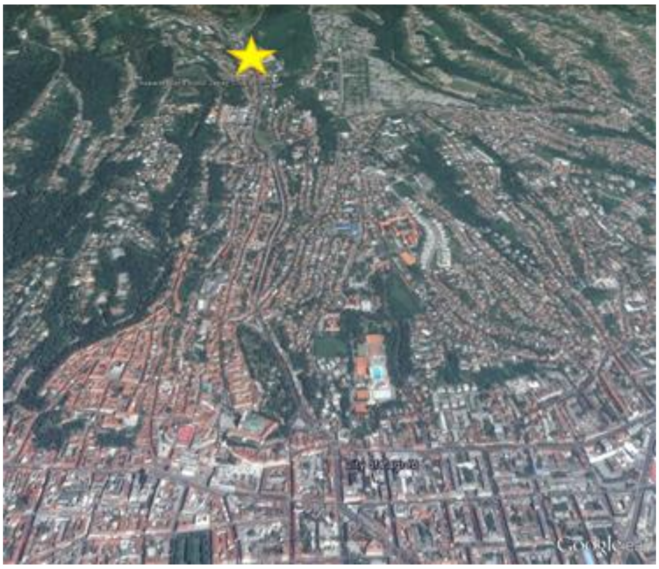

*The city of Zagreb and the sampling point (indicated on the map by the yellow star: the ZGR site). Source: Google Maps*

<u>Budapest – BDP.</u> The BDP site (ZGR: 47°25’40.35” N; 19°11’11.03” E) was set up in the Southside of the city of Budapest (1.732.000 inhabitants), in *Gilice Square* near the city center. The BDP site is representative of a typical urban background site.

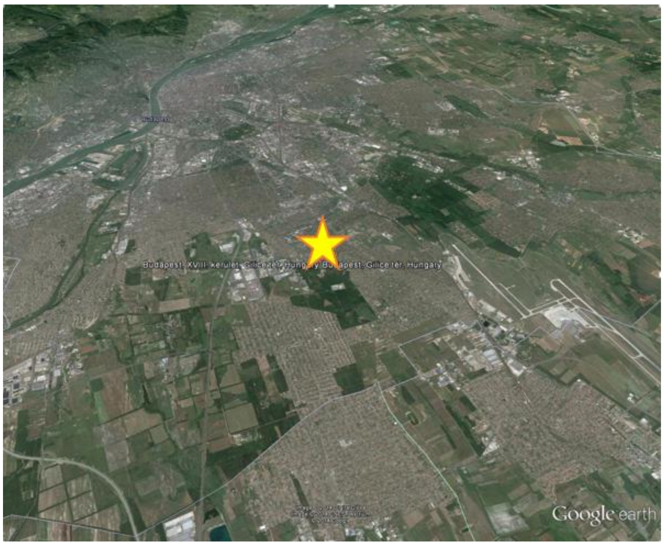

*The city of Budapest and the sampling point (indicated on the map by the yellow star: the BDP site). Source: Google Maps*

<u>Sofia- SOF.</u> The SOF site (ZGR: 42°39’19.08” N; 23°23’4.92” E) was set up in the Southwest side of the city of Sofia (1.211.000 inhabitants). The SOF site is an urban background site, located about 6 km away from the city center.

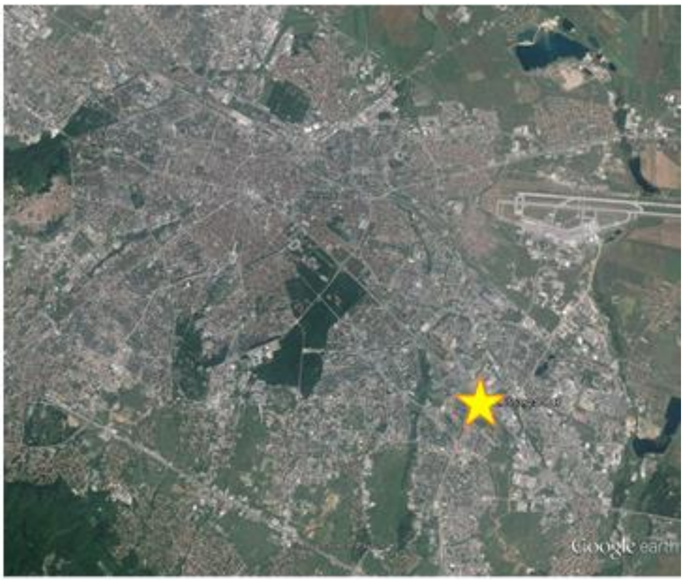

*The city of Sofia and the sampling point (indicated on the map by the yellow star: the SOF site). Source: Google Maps*

## S2. PM chemical analysis

| | Zagreb (ZGR) | | | Budapest (BDP) | | | Sofia (SOF) | | |
|---|---|---|---|---|---|---|---|---|---|
| | MDL ($\mu g\ m^{-3}$) | *Analytical uncertainty (%)* | Method | MDL ($\mu g\ m^{-3}$) | *Analytical uncertainty (%)* | Method | MDL ($\mu g\ m^{-3}$) | *Analytical uncertainty (%)* | Method |
| **PM10** | | | | | | | 0 | ***5*** | gravimetric |
| **PM2.5** | 1 | ***8,4*** | gravimetric | 0,5 | ***5*** | Beta-ray | | | |
| **PM1** | | | | | | | | | |
| **OC** | 0,18 | ***6,8*** | TOT | 0,1 | ***5*** | TOT | | | |
| **EC** | 0,01 | ***14,6*** | TOT | 0,01 | ***5*** | TOT | | | |
| **$SO_4^{2-}$** | 0,008 | ***16,5*** | IC | 0,003 | ***5*** | IC | 0 | ***5*** | IC |
| **$NO_3^-$** | 0,13 | ***16,5*** | IC | 0,003 | ***5*** | IC | 0 | ***5*** | IC |
| **$Cl^-$** | 0,05 | ***16,5*** | IC | 0,003 | ***5*** | IC | 0 | ***5*** | IC |
| **$NH_4^+$** | | | | 0,001 | ***5*** | photometry | 0,075 | ***5*** | IC |
| **$Na^+$** | | | | 0,002 | ***5*** | AAS | 0,059 | ***5*** | IC |
| **$K^+$** | | | | 0,001 | ***5*** | AAS | 0,002 | ***5*** | IC |
| **$Ca^{2+}$** | | | | 0,001 | ***5*** | AAS | 0,012 | ***5*** | IC |
| **$Mg^{2+}$** | | | | 0,001 | ***5*** | AAS | 0,003 | ***5*** | IC |
| **Ag** | 0,00038 | ***4,3*** | ED-XRF | | | | 4E-04 | ***8*** | ED-XRF |
| **Al** | | | | 0,0054 | ***8*** | PIXE | | | |
| **As** | | | | | | | 0,008 | ***8*** | ED-XRF |
| **Ba** | 0,00019 | ***50*** | ED-XRF | 0,0026 | ***8*** | PIXE | 2E-04 | ***8*** | ED-XRF |
| **Br** | 0,00068 | ***24,6*** | ED-XRF | 0,0017 | ***8*** | PIXE | 7E-04 | ***8*** | ED-XRF |
| **Ca** | 0,0005 | ***50*** | ED-XRF | 0,0032 | ***8*** | PIXE | 5E-04 | ***8*** | ED-XRF |
| **Cd** | | | | | | | 4E-04 | ***8*** | ED-XRF |
| **Cl** | | | | 0,0018 | ***8*** | PIXE | 0,003 | ***8*** | ED-XRF |
| **Co** | 0,00055 | ***29,9*** | ED-XRF | 0,002 | ***8*** | ED-XRF | | | |
| **Cr** | 0,0009 | ***50*** | ED-XRF | 0,0015 | ***8*** | ED-XRF | 9E-04 | ***8*** | ED-XRF |
| **Cs** | 0,00019 | ***38,0*** | ED-XRF | | | | | | |
| **Cu** | 0,00095 | ***4,2*** | ED-XRF | 0,0009 | ***8*** | PIXE | 1E-03 | ***8*** | ED-XRF |
| **I** | | | | | | | 3E-04 | ***8*** | ED-XRF |
| **K** | 0,00075 | ***2,5*** | ED-XRF | 0,0044 | ***8*** | PIXE | 8E-04 | ***8*** | ED-XRF |
| **Fe** | 0,00955 | ***0,8*** | ED-XRF | 0,0045 | ***8*** | PIXE | 0,01 | ***8*** | ED-XRF |
| **Ge** | 0,00095 | ***54,3*** | ED-XRF | | | | | | |
| **La** | 0,00019 | ***14,7*** | ED-XRF | | | | | | |
| **Mn** | 0,00061 | ***10,3*** | ED-XRF | 0,0008 | ***8*** | PIXE | 6E-04 | ***8*** | ED-XRF |
| **Ni** | 0,00065 | ***50*** | ED-XRF | 0,0005 | ***8*** | PIXE | 7E-04 | ***8*** | ED-XRF |
| **P** | | | | | | | 0,004 | ***8*** | ED-XRF |
| **Pb** | 0,00084 | ***33,5*** | ED-XRF | 0,0028 | ***8*** | PIXE | 8E-04 | ***8*** | ED-XRF |
| **Rb** | 0,0005 | ***16,7*** | ED-XRF | 0,0005 | ***8*** | ED-XRF | 5E-04 | ***8*** | ED-XRF |
| **S** | 0,00528 | ***12,6*** | ED-XRF | 0,0078 | ***8*** | PIXE | 0,005 | ***8*** | ED-XRF |
| **Sb** | | | | | | | 4E-04 | ***8*** | ED-XRF |
| **Sc** | 0,00095 | ***47,6*** | ED-XRF | | | | | | |
| **Se** | | | | 0,0005 | ***8*** | ED-XRF | | | |
| **Si** | | | | 0,0039 | ***8*** | PIXE | | | |
| **Sn** | | | | | | | 2E-04 | ***8*** | ED-XRF |
| **Sr** | 0,0005 | ***9,6*** | ED-XRF | 0,0005 | ***8*** | ED-XRF | 5E-04 | ***8*** | ED-XRF |
| **Ti** | 0,0005 | ***8,3*** | ED-XRF | 0,0007 | ***8*** | PIXE | 5E-04 | ***8*** | ED-XRF |
| **V** | 0,0003 | ***6,2*** | ED-XRF | 0,0007 | ***8*** | PIXE | 5E-04 | ***8*** | ED-XRF |
| **Zn** | 0,00056 | ***5,4*** | ED-XRF | 0,0027 | ***8*** | PIXE | 6E-04 | ***8*** | ED-XRF |
| **Zr** | | | | | | | 4E-04 | ***8*** | ED-XRF |
| **BaP** | 0,00001 | ***7,8*** | HPLC-FLD | | | | | | |
| **BhiP** | 0,00001 | ***7,8*** | HPLC-FLD | | | | | | |
| **IcdP** | 0,00002 | ***7,8*** | HPLC-FLD | | | | | | |
| **LEVO** | | | | 0,0002 | ***3*** | GC-MS | | | |

*Table S1. Minimum detection limit (MDL; $\mu g\ m^{-3}$) and analytical uncertainty (%) provided for all analyzed compounds in Zagreb (ZGR), Budapest (BDP) and Sofia (SOF).*

## S3. Reconstruction of OC/EC missing data in PM2.5 of Zagreb

The OC/EC data in PM samples of Zagreb were collected during the sampling period (all year, 2013), but OC/EC data in $PM_{2.5}$ were missing due to technical reasons for two and a half months (since 01 January to 14 March 2013, 73 days: ~20% of the PM sampling data). In Zagreb, OC/EC were analysed on 24h samples for both $PM_{2.5}$ and in $PM_{10}$, and there were not missing data for $PM_{10}$. To make available the information of the carbonaceous fraction in a large number of $PM_{2.5}$ samples for the PMF analysis, we estimated the OC missing concentrations in $PM_{2.5}$ from the OC concentration in $PM_{10}$ for the same site and same days. To that end we calculated a regression curve between OC data in $PM_{2.5}$ and $PM_{10}$ samples in Zagreb in the days of the year when OC was simultaneously analysed in both PM fractions (15 March-31 December, 292 days). The parameters of the regression curve between OC($PM_{2.5}$) and OC($PM_{10}$) ($R^2$=0.95; slope = 0.86, intercept = 0.06) were used to estimate daily OC missing concentrations in $PM_{2.5}$ for the time window 01 January-14 March 2013 (Figure S1A).
The correlation between EC($PM_{2.5}$) and EC($PM_{10}$) was weaker ($R^2$=0,63) (Figure S1A). Therefore, EC missing concentrations in $PM_{2.5}$ were estimated using the EC obtained from the light absorption coefficient α determined in the same days and same site which was better correlated with EC ($R^2$ = 0.73; slope = 0.61, intercept = 0.05) (Figure S1B).

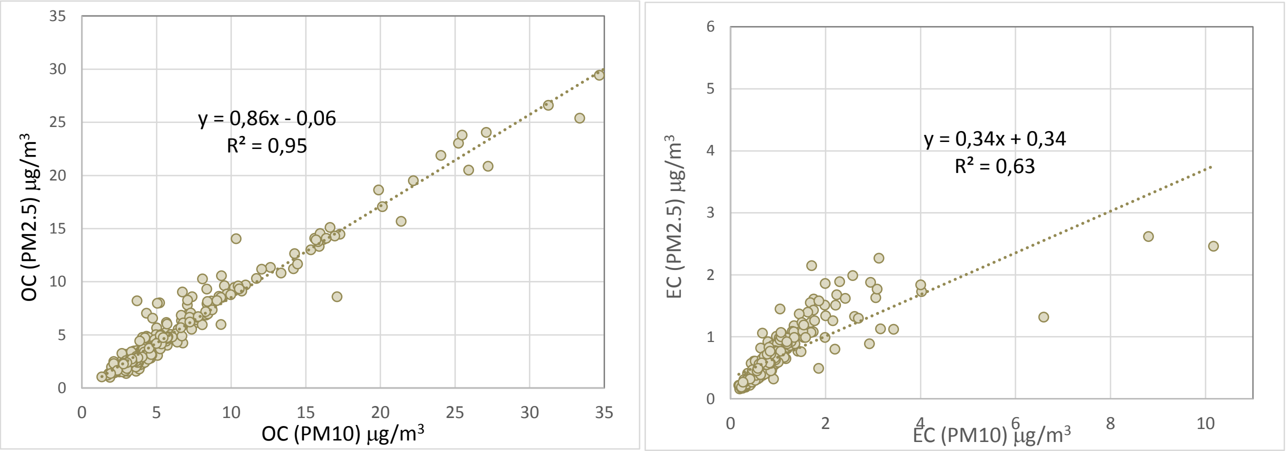


*Figure S1A. Correlation between OC data in PM2.5 and PM10 samples, and EC data in PM2.5 and PM10 samples of Zagreb (Croatia). Period: 15 March-31 December 2013 (n=292).*

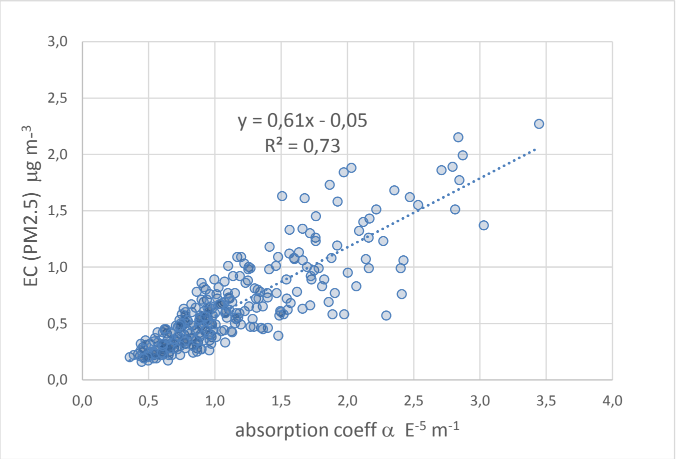


*Figure S1B. Correlation between EC data ($\mu g\ m^{-3}$) in PM2.5 and absorption coefficient $\alpha$ ($E^{-5}\ m^{-3}$) in Zagreb (Croatia). Period: 15 March-31 December 2013 (n=292).*

## S4. PMF analysis

| Diagnostic | ZGR-COLD | ZGR-WARM | BDP | SOF |
|---|---|---|---|---|
| **Factors** | 5 (1-SEC; 2-BB; 3-SOIL; 4-TR; 5-IND) | | 5 (1-SEC; 2-BB; 3-NITRATE+BB; 4-SOIL; 5-TR) | 6 (1-SEC; 2-BB; 3-SOIL: 4-TR; 5-FUEL OIL; 6-IND1 Lead) |
| **$Q_{expected}$** | 1665 | 1223 | 1310 | 894 |
| **$Q_{robust}$** | 3344,9 | 2578,4 | 2010,2 | 2144,6 |
| **$Q_{true}$** | 3375,7 | 2539,2 | 2015,8 | 2142,0 |
| **$Q_{true}/Q_{robust}$** | 1,01 | 1,01 | 1,00 | 1,00 |
| **$Q_{robust}/Q_{expected}$** | 2,03 | 2,08 | 1,53 | 2,39 |
| **Species with $Q/Q_{expected}$ > 3*** | Mn, $NO_3^-$ | $NO_3^-$ | Cl, Cr | Ti, V, Mn, Cu, Cl-, $NO_3^-$ |
| **DISP %dQ** | 0 | -0,017% | 0 | 0,50% |
| **DISP swaps** | 0 | 0 | 0 | 0 |
| **BS mapping** | Factor 3-SOIL 77%; Factor 5-IND 93%, Factor 4-TR 99%; 100% all other factors | Factor 1-SEC 98%; Factor 2-BB factor 85%; 100% all other factors | Factor 1-SEC 97% Factor 2-BB 87%; Factor 3-NITRATE RICH+BB 93%; 100% all other factors | Factor 6-IND 89%; ≥99% all other factors |

*Table S2. Summary of the PMF output and EE diagnostics.*

| Zagreb (ZGR) PM2.5 - Source contribution estimation (SCE) $\mu g\ m^{-3}$ | | | | | | | | | | |
|---|---|---|---|---|---|---|---|---|---|---|
| **Factor/Source** | **whole period** 2013 (1 Jan-31 Dec) n= 335 | | **winter (W)** 21 Dic-20 March n= 85 | | **spring (SP)** 21 March-20 June n= 82 | | **summer (SU)** 21 June-20 Sept n= 80 | | **fall (F)** 21 Sept-20 Dic n= 88 | |
| | **AV** | ***SD*** | **AV** | ***SD*** | **AV** | ***SD*** | **AV** | ***SD*** | **AV** | ***SD*** |
| Secondary aerosol **(SEC)** | 7.5 | *9.2* | 10.5 | *11.8* | 6.5 | *7.6* | 5.5 | *3.8* | 7.4 | *10.5* |
| Biomass Burning **(BB)** | 8.1 | *12.1* | 16.9 | *14.4* | 2.8 | *3.7* | 1.6 | *1.3* | 10.4 | *14.2* |
| **Soil** | 1.0 | *1.5* | 0.7 | *0.6* | 1.7 | *2.6* | 0.8 | *1.0* | 0.8 | *0.8* |
| Traffic **(TR)** | 3.3 | *4.0* | 4.2 | *5.5* | 2.4 | *1.2* | 1.6 | *0.9* | 5.1 | *4.9* |
| Industry **(IND)** | 1.9 | *2.0* | 2.6 | *2.7* | 0.8 | *0.9* | 1.1 | *0.7* | 2.8 | *2.2* |

*Table S3. Source contribution estimate (SCE) ($\mu g\ m^{-3}$) to PM2.5 in Zagreb (ZGR). The average (AV) value with standard deviation (SD) for the whole sampling period and seasons.*

| Budapest (BDP) PM2.5 - Source contribution estimation (SCE) $\mu g\ m^{-3}$ | | | | | | |
|---|---|---|---|---|---|---|
| **Factor/Source** | **whole period** 2015 (09 Feb-18 May) n= 94 | | **winter (W)** 09 Feb- 20 March n= 37 | | **spring (SP)** 21 March-18 May n= 57 | |
| | **AV** | *SD* | **AV** | *SD* | **AV** | *SD* |
| Secondary aerosol **(SEC)** | 3.8 | *4.1* | 7.0 | *4.4* | 1.8 | *2.2* |
| Biomass Burning **(BB)** | 3.8 | *3.8* | 6.3 | *4.6* | 2.1 | *1.7* |
| Nitrate rich + BB | 2.5 | *4.1* | 5.1 | *5.3* | 0.8 | *1.5* |
| **Soil** | 3.2 | *3.8* | 2.9 | *5.3* | 3.5 | *2.4* |
| Traffic **(TR)** | 2.0 | *0.9* | 1.7 | *1.0* | 2.2 | *0.7* |

*Table S4. Source contribution estimate (SCE) ($\mu g\ m^{-3}$) to PM2.5 in Budapest (BDP). The average (AV) value with standard deviation (SD) for the whole sampling period and seasons.*

| Sofia (SOF) PM10 - Source contribution estimation (SCE) $\mu g\ m^{-3}$ | | | | | | | | |
|---|---|---|---|---|---|---|---|---|
| **Factor/Source** | **whole period** 2012-13 n= 99 | | **winter (W)** 06-25 Feb 12; 17 Dic 12-11 Feb 13 n= 47 | | **summer (SU)** 02-26 July 12; 01-23 July 13 n= 45 | | **fall (F)** 22-31 Oct 12 n=7 | |
| | **AV** | *SD* | **AV** | *SD* | **AV** | *SD* | **AV** | *SD* |
| Secondary aerosol **(SEC)** | 17,2 | *18,2* | 21,9 | *23,4* | 11,5 | *4,6* | 9,6 | *6,3* |
| Biomass Burning **(BB)** | 9,3 | *23,9* | 17,0 | *30,4* | 0,0 | *0,0* | 2,5 | *1,4* |
| **Soil** | 8,8 | *7,0* | 6,5 | *6,9* | 11,4 | *6,1* | 15,9 | *6,4* |
| Traffic **(TR)** | 1,7 | *1,9* | 2,1 | *2,2* | 1,2 | *1,4* | 2,9 | *2,3* |
| **Fuel Oil** - Ba,V rich | 2,3 | *3,5* | 3,8 | *4,1* | 0,6 | *1,3* | 7,3 | *4,3* |
| Industry **(IND)** - Pb rich | 2,0 | *3,4* | 3,3 | *4,1* | 0,4 | *0,9* | 0,6 | *1,0* |

*Table S5. Source contribution estimate (SCE) ($\mu g\ m^{-3}$) to PM10 in Sofia (SOF). The average (AV) value with standard deviation (SD) for the whole sampling period and seasons (fall is considered to be poorly represented due to the limited number of samples; n=7).*

## S4.1 Factors associated to BB in the PMF analysis of PM 2.5 in BDP

Two factors were associated to biomass burning (BB) source in the PMF analysis of PM2.5 in Budapest (BDP). Both of them are characterized by the presence of specific BB source markers, i.e. LEVO that is a unique BB tracer and other markers for this source such as K, Zn, Cl, OC (Figure 3A). The two factors identified as BB source differ in their content of nitrate, that reaches 35% of the mass in the factor “Nitrate rich + BB” and is absent in the factor “BB”. However the most distinctive feature for these two factors are their time trends. The BB presents a rather typical seasonal trend for this source with higher contributions in winter that gradually decrease to achieve minimum levels in late spring. On the other hand, the Nitrate rich+BB factor is characterized by a distinct episode in the second half of February followed by minor isolated events in March.
In the BDP solution ammonium is almost totally (92%) allocated to “Secondary Aerosol (SEC)” as a counterion for secondary sulfate and to a lesser extent to nitrate. Very little or no ammonium is allocated to the other factors suggesting other counterions such as $K^+$, $Na^+$, $Ca^{2+}$, $Mg^{2+}$ or other cations (we did not analyse all) are the alkaline species that neutralize the nitrate acidity in the considered factor.

No significant changes occurred between the two factors “BB” and “Nitrate rich + BB” with respect to ratios like OC/LEVO, K/LEVO, and such ratios were comparable to typical ,although highly variable, ratios in BB source, as shown in Table S6.

| | Factor “BB” (this study, BDP) | Factor “Nitrate rich + BB” (this study, BDP) | BB source (from literature) |
|---|---|---|---|
| **OC/LEVO** | **12.9** | **12.2** | 7.35 (1) ;<br>12.2 (2)<br>8.9±7.6 (3) |
| **LEVO/K** | **2.1** | **1.3** | 0.10±0.00 wheat straw;<br>0.21±0.08 wheat straw;<br>23.96±1.82 pine wood;<br>5.89±0.53 poplar wood (4)<br>1.9-5.7 (5) |

*(1) OC/LEVO for BB source at background sites in Europe (Puxbaum et al., 2007)*
*(2) OC/LEVO from PMF derived BB profiles (Piazzalunga et al., 2011)*
*(3) OC/LEVO for BB source category (SPECIEUROPE)*
*(4) LEVO/$K^+$ in various BB emissions (Cheng et al., 2013)*
*(5) LEVO/K for BB source category (SPECIEUROPE)*

*Table S6. OC/LEVO and K/LEVO ratios in the factor BB and Nitrate rich + BB in Budapest (BDP) compared with literature data for the BB source profile*

The split of biomass burning in two factors is unlikely to be an artifact (artificial split of one source in two factors) because in addition to the model diagnostics (Table S2) is supported by distinct time trends that are also associated with different geographical patterns, as shown by the analysis of backward trajectories (Section 3.4.2 of the paper).

## S5. CPF results

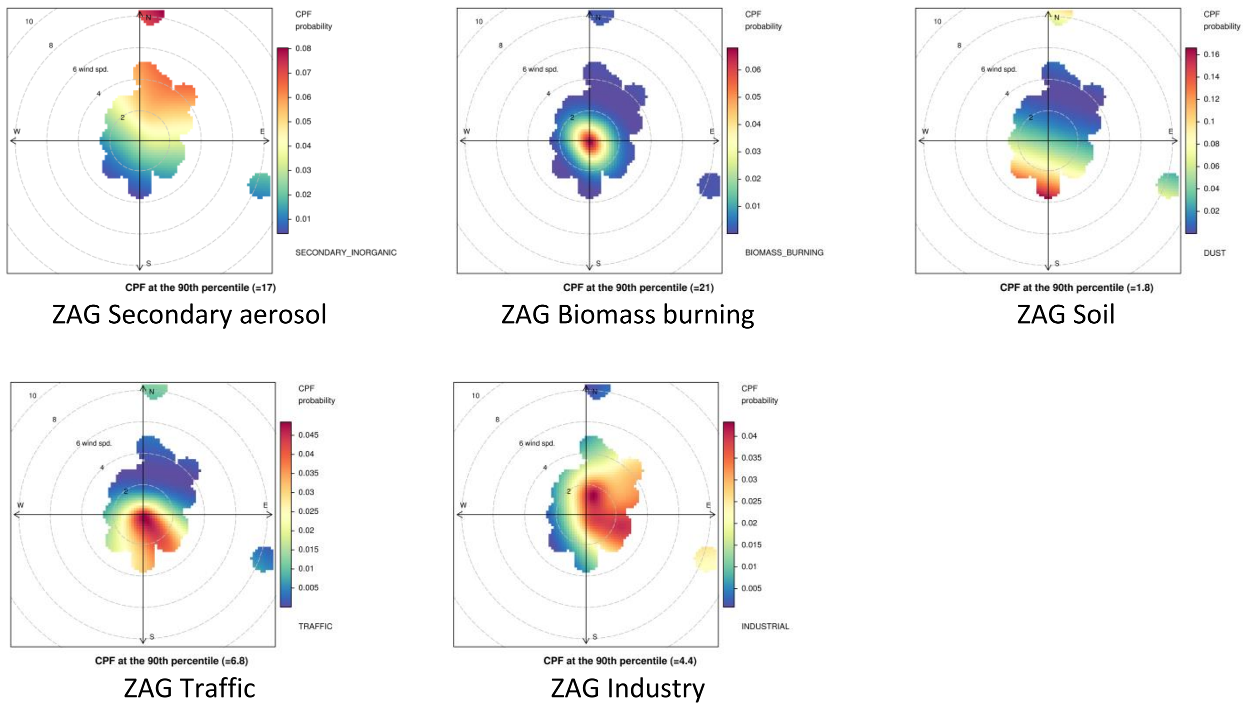


*Figure S2. Zagreb, conditional probability function (CPF) (all sources)*

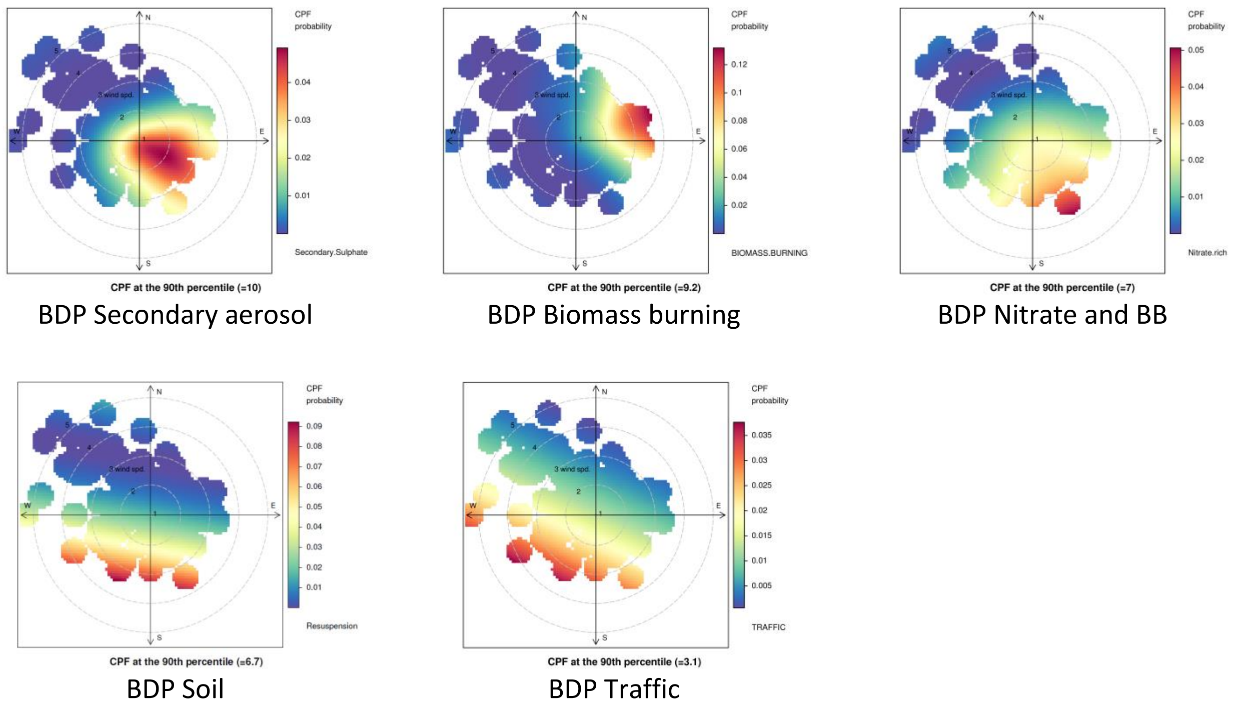


*Figure S3. Budapest, conditional probability function (CPF) (all sources)*

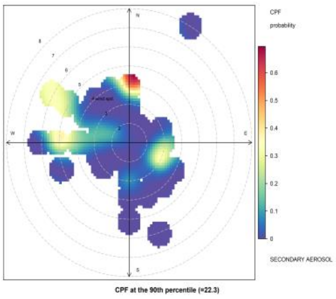


SOF Secondary aerosol

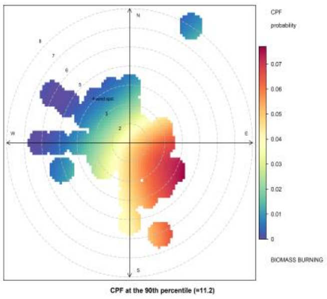


SOF Biomass burning

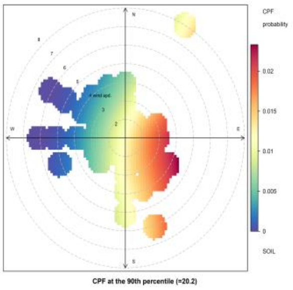


SOF Soil

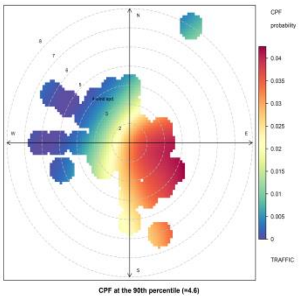


SOF Traffic

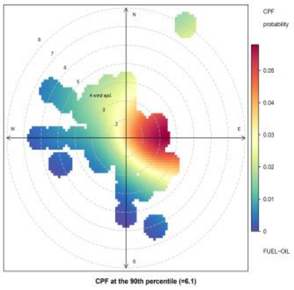


SOF Fuel oil

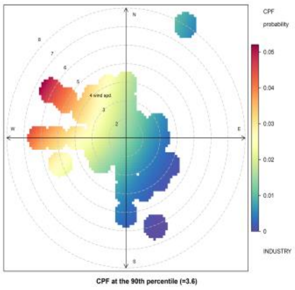


SOF Industry Pb rich

*Figure S4. Sofia, conditional probability function (CPF) (all sources)*

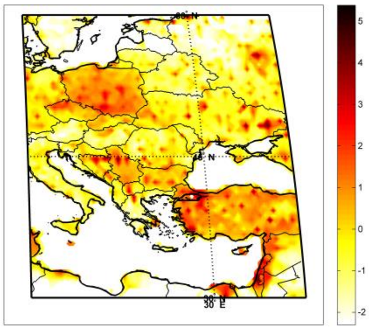

$SO_2$ emissions LAND

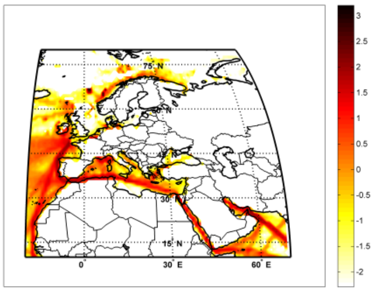

$SO_2$ emissions SHIPS

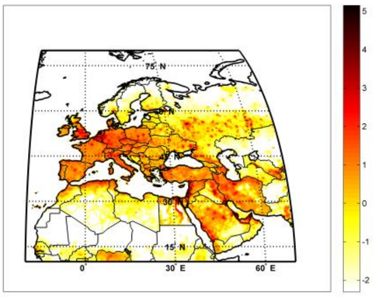

$NO_X$ emissions

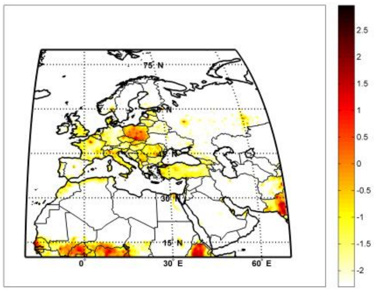

$PM_{10}$ residential emissions

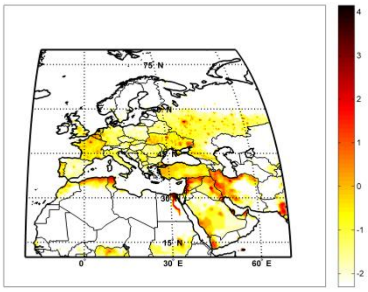

$PM_{10}$ industrial emissions

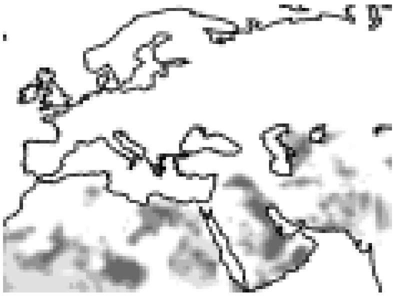

Dust emission areas

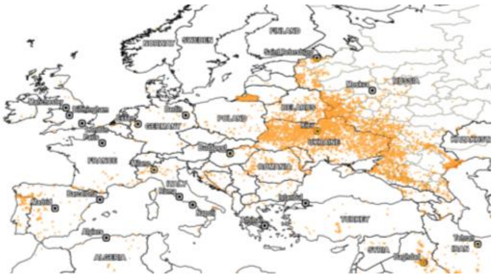

Agricultural fires

*Figure S5. Emission maps (ECLIPSE EMISSIONS DATASET; reference year 2010 http://eclipse.nilu.no/ ), dust emission areas (TOMS satellite; Prospero et al., 2002) and agricultural fires in February – March 2015 (MODIS satellite).*

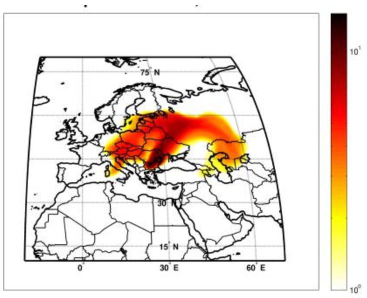

6th July 2012

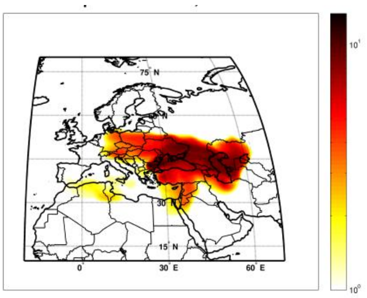

24th July 2012

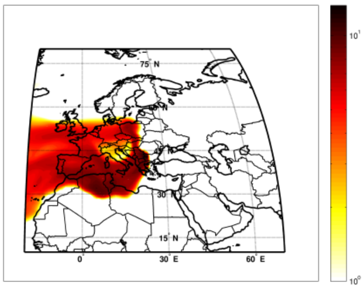

3rd February 2013

*Figure S6. Backward trajectories corresponding with SOIL episodes in Sofia*

## S6. Details on the PSCF analysis

### S6.1 The use of a binomial distribution to reduce the statistical noise

Various versions of PSCF have been proposed in previous publications (Dimitriou and Kassomenos, 2016; Polissar et al., 2001). Vasconcelos et al. 1996 suggested the use of a binomial distribution in order to test the significance of PSCF and to reduce the statistical noise. In this study PSCF was applied in the following manner:

Since the PSCF is computed as a ratio of the counts of selected events ($m_{ij}$) to the counts of all events ($n_{ij}$), it is likely that relatively small ($n_{ij}$), which are often related to sparse trajectory coverage of the more distant grid cells, may result in PSCF with high uncertainty in the apparent high value. First we set a criterion for the residence time value in a cell that has low uncertainty, so as to keep a grid cell in the PSCF analysis. We set this value (p) equal to the average residence time from all cells (n), after we have excluded the cells that have residence time equal to zero. In other words, the number of seconds (residence time, s) in each grid cell is compared to the average of residence times in seconds from all cells with $s>0$ (n), using a binomial test. The result of the binomial test is the probability of observing the value of s in a cell, while the actual value of the cell is p, and therefore significant. For example, if this probability is lower than 1%, there is very low probability that this cell will actually have a residence time equal to p, therefore it will not be considered a significant cell, and its PSCF result is decreased by a weighting factor. These weighting function limit values were obtained empirically by running the PSCF program many times and applying the trial and error method. Figure S7 shows an example of 5 days PSCF analysis with and without the binomial filter for sources affected by long-range transport in Budapest. When we do not apply the filter for the Nitrate rich source, we observe high PSCF values in the Arctic. For the Biomass Burning source, the very distant sources indicated over Cyprus, Turkey and the Caspian Sea are weighted down to a more reasonable probability (there is no evidence to support a contribution to BB from such a distant location).

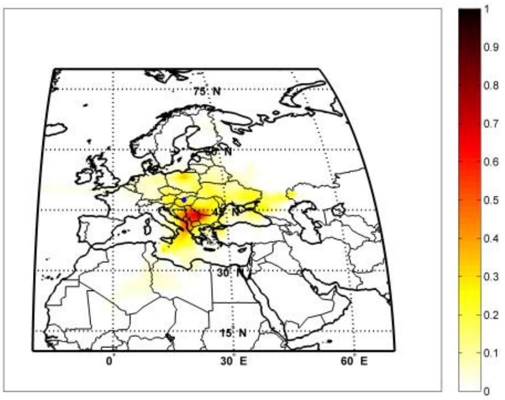


**BDP** Secondary aerosol (5 days),
90th percentile, binomial filter

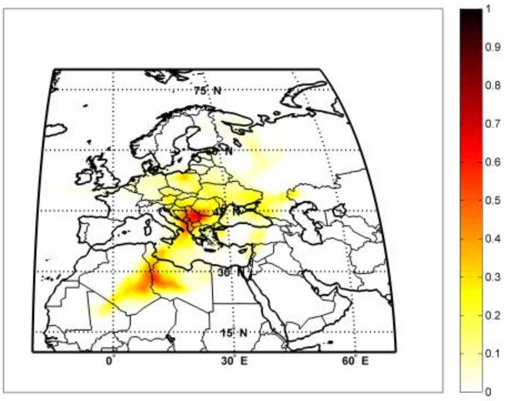


**BDP** Secondary aerosol (5 days),
90th percentile, no filter.

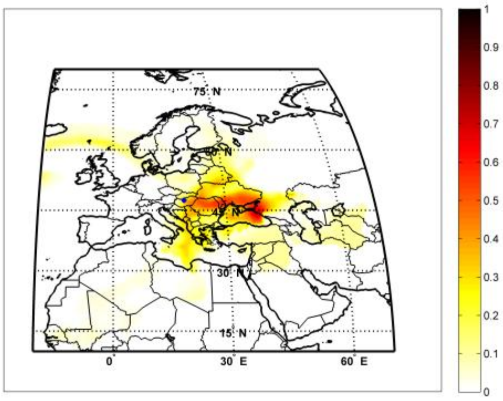


**BDP** Biomass burning (5 days),
90th percentile, binomial filter

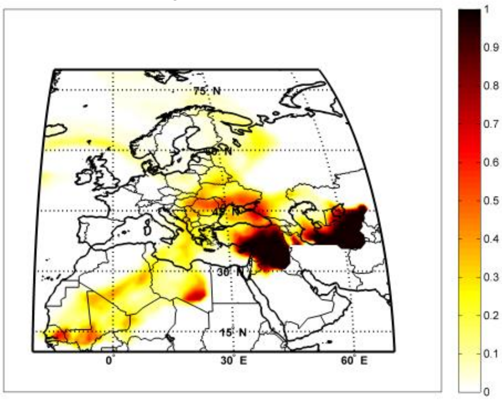


**BDP** Biomass burning (5 days),
90th percentile, no filter.

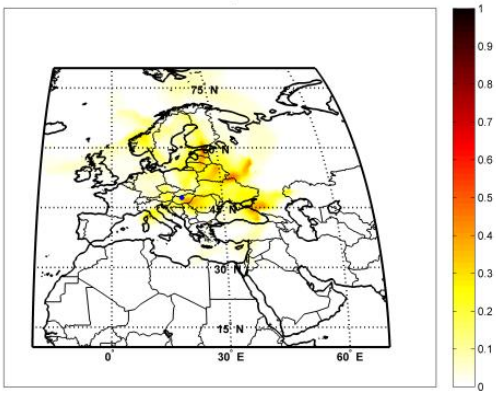


**BDP** Nitrate rich and BB (5 days),
90th percentile, binomial filter

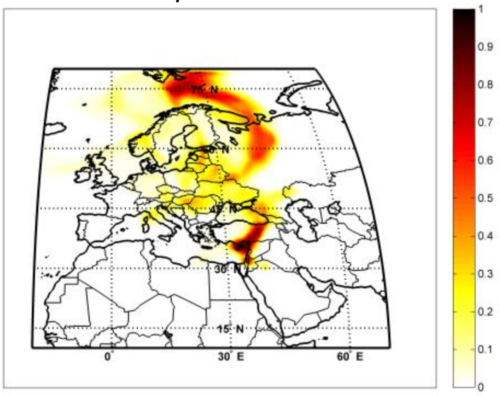


**BDP** Nitrate rich and BB (5 days),
90th percentile, binomial filter.

*Figure S7. Biomass Burning, Nitrate rich and BB, secondary aerosol source in Budapest (BDP), 5 days PSCF at the 90th percentile, with and without the binomial filter.*

**S6.2 Altitude of the trajectories.**

“There is a limit to the height above a station that can be used when running Lagrangian models, because at night time the Boundary Layer Height (BLH) might be very low, leading to different PM concentrations at the trajectory height from the ones measured at the station. For Sofia, during 2012-2015, ECMWF reanalysis ERA-interim data indicate that in only 5% of the 3hr intervals BLH is lower than 300 m. FLEXPART is particularly suitable to minimize the errors deriving from the height because it works with statistical distributions rather than deterministic values. The model was set to release a high number (20,000) of computational particles (finite air masses) every hour, and thus it covers a broad range of probabilities in atmospheric circulation. FLEXPART simulates very well air mass transport in the Mesoscale (Brioude et al., 2012).

Finally, it is important to bear in mind that PSCF is a tool to provide information on impact of distant sources to our area of interest and not high concentrations induced by local effects. When a long dataset is available the paths of transport during local events are random leading to no statistically significant results that are consequently removed by the binomial filter described above.”

## S6.3 Multi-site PSCF analysis for the Danube region

A multi site PSCF analysis was applied for the sources that had a significant probability to be transported in the mesoscale. The results indicate that there are two main source areas for the Soil aerosol (North Africa, Caspian Sea region). There is also a source indicated in Asia Minor. The combined Secondary aerosol results indicate that the main source areas are in the European Turkey region, and the St Petersburg region on the North-East.

| | |
|---|---|
| 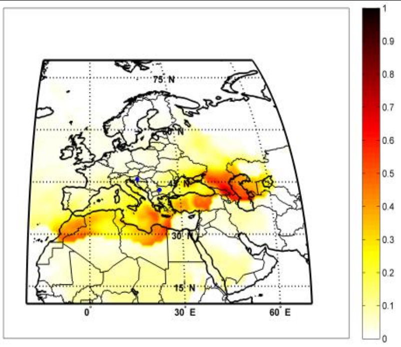  | 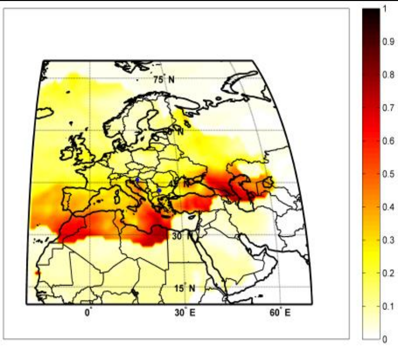  |
| Combined Soil aerosol PSCF analysis for Sofia, Zagreb at the 90$^{th}$ percentile, 10 days backward run. | Combined Soil aerosol PSCF analysis for Sofia, Zagreb at the 75$^{th}$ percentile, 10 days backward run. |
| 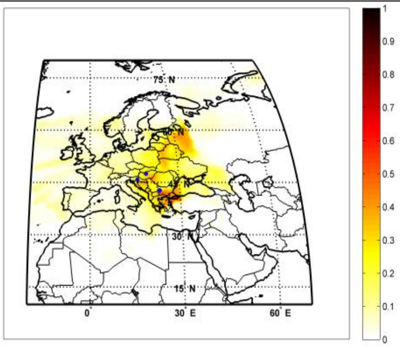  | 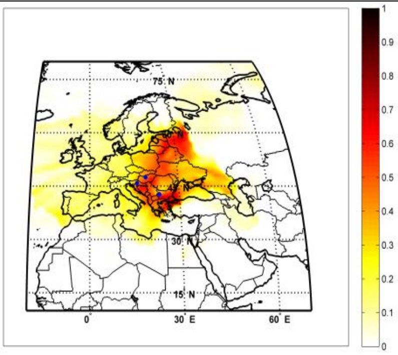  |
| Combined Secondary aerosol PSCF analysis for Sofia, Zagreb, and Budapest at the 90$^{th}$ percentile, 5 days backward run. | Combined Secondary aerosol PSCF analysis for Sofia, Zagreb, and Budapest at the 75$^{th}$ percentile, 5 days backward run. |

*Figure S8. Multi-site PSCF analysis for the Danube region based on results from Sofia, Zagreb and Budapest (Secondary aerosol) and Sofia, Zagreb (Soil). 75$^{th}$ and 90$^{th}$ percentile.*

In order to apply PSCF, for each cell we calculate the ratio $PSCF_{ij} = m_{ij}/n_{ij}$. $m_{ij}$ is the number of seconds in a cell corresponding to the measurements that have concentration higher than the 90th or 75th percentile of the estimated source concentration, and $n_{ij}$ is the total number of seconds (residence time) in a cell for all measurements.

$PSCF_{ij}$ is the measure of probability of a 1°x1° grid cell to contribute to the injected mass which is from there on transported and is related to the mass concentration measured at the receptor sites considered.

The methodology used in order to apply multi-site PSCF was similar to the one reported by (Han et al., 2007). The results based on the 90$^{th}$ and 75$^{th}$ percentile for each city were used in the following equation:

$$\left(PSCF\_Multi\_Site_{ij}\right) = \frac{\left(m_{ij}\_Sofia\right) + \left(m_{ij}\_Budapest\right) + \left(m_{ij}\_Zagreb\right)}{\left(n_{ij}\_Sofia\right) + \left(n_{ij}\_Budapest\right) + \left(n_{ij}\_Zagreb\right)}$$

$m_{ij}$_*Sofia(Budapest, Zagreb)* = Time period (seconds) in a geographical cell (1X1 deg) where the air mass of a backward run has been present, corresponding to the measurements that have at this starting location concentration higher than the 90th or 75th percentile of the estimated selected source contribution to the PM concentration in Sofia (Budapest, Zagreb).

$n_{ij}$_*Sofia(Budapest, Zagreb)* = Sum of time periods (seconds) i.e. residence time, spent in a geographical cell by air masses of all 10 day backward runs corresponding to the 24h sampling periods in Sofia (Budapest, Zagreb).

## S6.4 Influence of a different percentile ($75^{th}$) threshold for the PSCF analysis in the Danube region

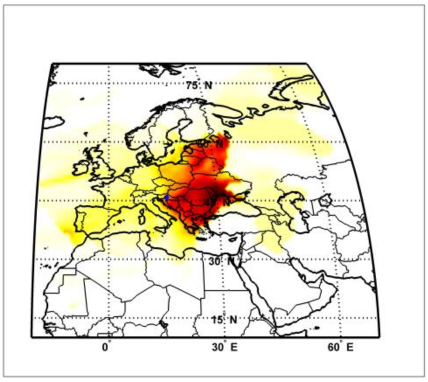


**ZAG** Secondary aerosol at the $75^{th}$ percentile (5 days)

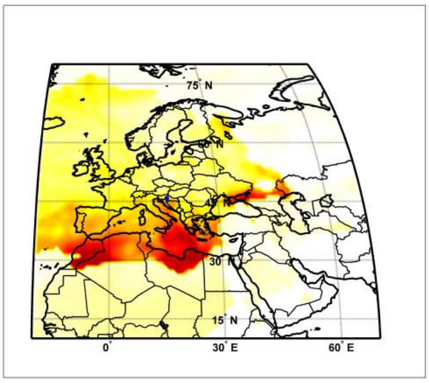


**ZAG** Soil at the $75^{th}$ percentile (10 days)

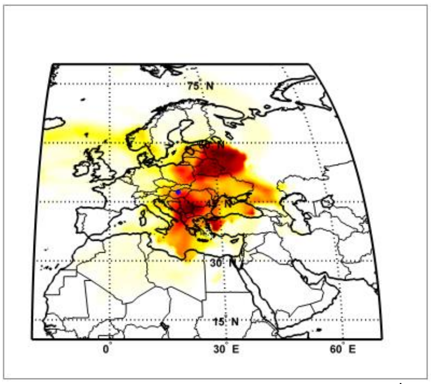


**BDP** Secondary aerosol at the $75^{th}$ percentile (5 days)

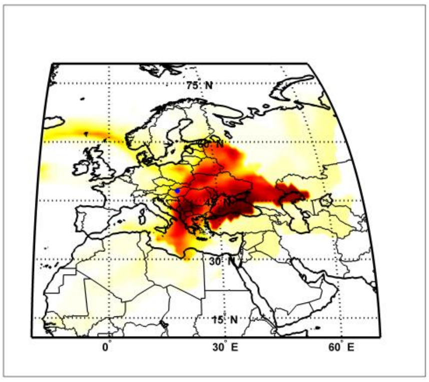


**BDP** Biomass burning at the $75^{th}$ percentile (5 days)

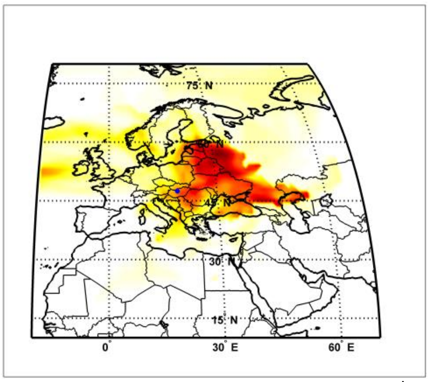


**BDP** Nitrate rich and BB at the $75^{th}$ percentile (5 days)

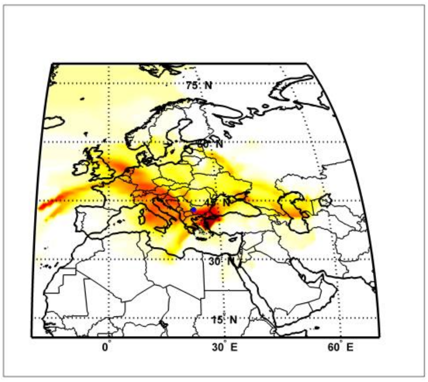


**SOF** Secondary aerosol at the $75^{th}$ percentile (5 days)

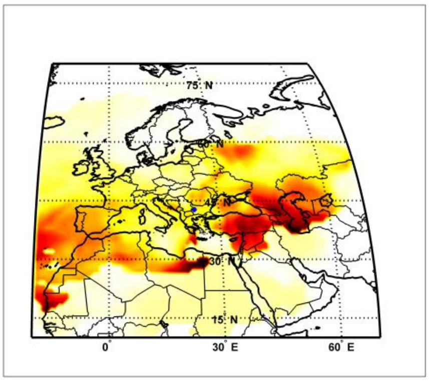


**SOF** Soil at the $75^{th}$ percentile (10 days)

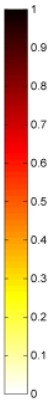


**probability**

*Figure S9. PSCF analysis for the Danube region at the $75^{th}$ percentile.*

The methodology followed was from (Uria-Tellaetxe et al., 2014), where the 75th and 90th percentiles are used. Also, at the Openair project, the 90th percentile is the default value for PSCF analysis (Carslaw and Ropkins, 2012).

We observe from the comparison of the above Figure S9 and Figure 6 of the manuscript, that on the 90th percentile the result is much more focused on source areas. For the Budapest Nitrate rich aerosol (aged Biomass Burning) the area between Moscow and Ukraine (further from the

measurement site) can be identified for the 90th percentile, while for the 75th percentile areas from smaller distances seem to contribute. For the Budapest fresh Biomass Burning aerosol, at the 90th percentile we observe that source areas are those that are close to Budapest (Romania, Ukraine), while at the 75th percentile, very distant areas seem to contribute to fresh aerosol (See also Figure S5 in Supplemental). Overall, the result for the 75th percentile is that it is diffused in comparison to that of the 90th percentile.

Our interpretation is that a lower threshold (75th Percentile in this case) to encompass a higher number of samples leads to the inclusion in the PSCF analysis of many samples where the contribution of the studied source is not clearly dominant. The results, therefore, represent a wider range of situations leading to higher spread of the source areas that not necessarily contribute to a better representation of the pollution origin. According to this test, the 90th percentile suggested in the literature seems to be appropriate for the purposes of our study.

## References


Brioude, J, Angevine, W M, McKeen, S A, Hsie, E Y. Numerical uncertainty at mesoscale in a Lagrangian model in complex terrain. Geoscientific Model Development 2012; 5; 1127-1136.

Carslaw, D C, Ropkins, K. Openair — an R package for air quality data analysis. Environmental Modelling & Software 2012; 27-28; 52–61.

Cheng, Y, Engling, G, He, K-B, Duan, F-K, Ma, Y-L, et al. Biomass source contribution to Bejing aerosol. Atmospheric Chemistry and Physics 2013; 13; 7765-7781.

Dimitriou, K and Kassomenos P. Combining AOT, Angstrom Exponent and PM concentration data, with PSCF model, to distinguish fine and coarse aerosol intrusions in Southern France. Atmospheric Research 2016; 172; 74-82.

Han Young-Ji, Thomas M. Holsen, Philip K. Hopke, Estimation of source locations of total gaseous mercury measured in New York State using trajectory-based models, In Atmospheric Environment, Volume 41, Issue 28, 2007, Pages 6033-6047, ISSN 1352-2310.

Latella, A., Stani, G., Cobelli, L., Duane, M., Junninen, H., Astorga, C., Larsen, B.R. Semicontinuous GC analysis and receptor modelling for source apportionment of ozone precursor hydrocarbons in Bresso, Milan, 2003, Journal of Chromatography A 2005; 1071 (1-2), pp. 29-39.

Karthikeyan, S and Balasubramanian, R. Determination of water-soluble inorganic and organic species in atmospheric fine particulate matter. Microchemical Journal 2006; 82; 49–55.

Piazzalunga, A, Belis, C, Bernardoni, V, Cazzulli, O, Fermo, P, Valli, G, Vecchi, R. Estimates of wood burning contribution to PM by the macro-tracer method using tailored emission factors. Atmospheric Environment 2011; 45; 6642-6649.

Polissar, A V, Hopke, P K, Harris, J M. Source regions for atmospheric aerosol measured at Barrow, Alaska. Environmental Science & Technology 2001; 35; 4214 – 4226.

Prospero, J. M., P. Ginoux, O. Torres, S. E. Nicholson, and T. E. Gill, Environmental characterization of global sources of atmospheric soil dust identified with the Nimbus 7 total ozone mapping spectrometer (TOMS) absorbing aerosol product Rev. Geophys. 2002; 40(1), 1002.

Puxbaum, H, Caseiro, A, Sanchez-Ochoa, A, Kasper-Giebl, A, Clayes, M, et al. Levoglucosan levels at background sites in Europe for assessing the impact of biomass combustion on the European aerosol background. Journal of Geophysical Research 2007; 112; D23S05.

SPECIEUROPE. http://source-apportionment.jrc.ec.europa.eu/Specieurope/index.aspx

Uria-Tellaetxe I. and Carslaw D., Conditional bivariate probability function for source identification, Environmental Modelling & Software, 2014, Volume 59, 1-9.

Vasconcelos, L A P, Kahl, J D W, Liu, D, Macias, E S, White, W H. Spatial resolution of a transport inversion technique. Journal of Geophysical Research 1996; 101; 19337-19342.
